# Correction: Learning Gene Networks under SNP Perturbations Using eQTL Datasets

**DOI:** 10.1371/journal.pcbi.1003608

**Published:** 2014-04-09

**Authors:** The PLOS Computational Biology Staff

## Notice of Republication

This article was republished on March 21, 2014, to correct errors introduced into the equations during typesetting. Please download this article again to view the correct version. The originally published, uncorrected article and the republished, corrected article are provided here for reference.

In the article PDF, in the Materials and Methods section, there were errors in Equations (6), (9) and (10), and in the equations in the text below Equation (6), above Equation (10), and in the paragraph below Equation (10). In all these cases, T should be superscript for "matrix transpose".

## Supporting Information

File S1 Originally published, uncorrected article.
(PDF)

File S2 Republished, corrected article.
(PDF)
