# Supplementary material for: Learning Gene Networks under SNP Perturbations Using eQTL Datasets
Source: PLoS Comput Biol. 2014 Feb 27;10(2):e1003420. doi: 10.1371/journal.pcbi.1003420 (PMC3937098; doi:10.1371/journal.pcbi.1003420)
Supplement: Text S1 — Proof of convexity of sparse CGGM optimization problem. (PDF) [file pcbi.1003420.s001.pdf]

Text S1: Proof of Convexity of Sparse CGGM Optimization Problem

Proposition 1. *The optimization problem for learning a sparse CGGM given as below is convex:*

$$\operatorname*{argmin}_{\mathbf{\Theta_{yy}},\mathbf{\Theta_{xy}}} L(\mathbf{\Theta_{xy}},\mathbf{\Theta_{yy}};\mathbf{X},\mathbf{Y})+\lambda_1||\mathbf{\Theta_{xy}}||_1+\lambda_2||\mathbf{\Theta_{yy}}||_1, \quad (1)$$

where $L(\mathbf{\Theta_{xy}},\mathbf{\Theta_{yy}};\mathbf{X},\mathbf{Y}) = 1/2\,\mathrm{tr}\big(\mathbf{Y}\mathbf{\Theta_{yy}}\mathbf{Y}^T\big) + \mathrm{tr}\big(\mathbf{X}\mathbf{\Theta_{xy}}\mathbf{Y}^T\big) + \sum_i \log Z(\mathbf{\Theta_{xy}},\mathbf{\Theta_{yy}},\mathbf{x}^i)$ *is the negative log-likelihood of data.*

Proof. The L_1 penalty is convex and we only need to show that the negative data log-likelihood $L(\mathbf{\Theta_{xy}}, \mathbf{\Theta_{yy}};\mathbf{X},\mathbf{Y})$ in Eq. (1) is convex. To prove the convexity of $L(\mathbf{\Theta_{xy}},\mathbf{\Theta_{yy}};\mathbf{X},\mathbf{Y})$, we use the standard approach for proving the convexity of a function by showing that the second derivative of the given function is positive definite [30]. We notice that a CGGM is a special case of a log-linear model. In addition, it is a standard result that the second derivative of negative data log-likelihood for a log-linear model is an expected feature covariance matrix [29]. Since a covariance matrix is always positive definite, the negative data log-likelihood for CGGM is convex. □
