# Supplementary material for: Learning Gene Networks under SNP Perturbations Using eQTL Datasets
Source: PLoS Comput Biol. 2014 Feb 27;10(2):e1003420. doi: 10.1371/journal.pcbi.1003420 (PMC3937098; doi:10.1371/journal.pcbi.1003420)
Supplement: Text S2 — Learning algorithm for sparse CGGMs. (PDF) [file pcbi.1003420.s002.pdf]

Text S2: Learning Algorithm for Sparse CGGMs

In this section, we provide the details of the optimization algorithm for learning sparse CGGM parameters. Although the optimization problem for sparse CGGMs is convex, the main challenge for solving the optimization problem arises from the non-smoothness of the L_1 penalty function. In order to handle this challenge efficiently, we notice that the optimization problem for sparse CGGM consists of the smooth function of data log-likelihood and the non-smooth function of the L_1 penalty. We adopt a variant of accelerated proximal gradient algorithms, called a Nesterov's second method [31], that has been developed as a general-purpose algorithm for optimizing a convex function that consists of a smooth continuously-differentiable part and a non-smooth part [31–33]. Among many variants of accelerated proximal gradient methods, we choose to use the Nesterov's second method because it has the additional capability of ensuring $\mathbf{\Theta_{yy}}$ to be positive definite throughout the iterative optimization procedure.

An accelerated proximal gradient method extends a proximal gradient method with an additional step of acceleration in each iteration. The proximal gradient method generalizes the standard gradient descent method for optimizing a smooth function, to handle the additional non-smooth part of the objective function. The gradient descent step in each iteration of the gradient-descent method can be viewed as constructing a local quadratic approximation of the objective around the current parameter estimate and minimizing this local approximation to update the parameter estimate. The proximal gradient method augment this gradient step with a proximal mapping step that amounts to a soft-thresholding operation for the case of L_1 penalty. An accelerated proximal gradient method further extends the proximal gradient method with an additional acceleration step to speed up the convergence. The acceleration step adds a momentum to the gradient step by updating the parameter in each iteration based on the results from the two proceeding iterations, rather than updating based on the parameter estimate only from the previous iteration. Such an acceleration step has been shown to improve both theoretical and empirical convergence rates of proximal gradient methods significantly.

We present the full optimization procedure for learning sparse CGGM parameters $\mathbf{\Theta}_{\text{all}} = \begin{pmatrix} \mathbf{\Theta_{xy}} \\ \mathbf{\Theta_{yy}} \end{pmatrix}$ in Algorithm 1. $\mathbf{W} = \begin{pmatrix} \mathbf{W_{xy}} \\ \mathbf{W_{yy}} \end{pmatrix}$ and $\mathbf{U} = \begin{pmatrix} \mathbf{U_{xy}} \\ \mathbf{U_{yy}} \end{pmatrix}$ are auxiliary variables of the same dimension as $\mathbf{\Theta}_{\text{all}}$ that store the intermediate values of the parameters in each iteration. Steps 1 and 2 of Algorithm 1 correspond to acceleration steps, where the amount of momentum δ_t for acceleration in iteration t is updated in Step 1 and the acceleration is performed on the intermediate variable $\mathbf{W}$ in Step 2 using δ_t. Step 3, in preparation for the proximal gradient step in Step 4, computes the negative data log-likelihood based on the current estimate of the parameters and its gradients given as:

$$\nabla L(\mathbf{\Theta}_{\text{all}}; \mathbf{X}, \mathbf{Y}) = \begin{pmatrix} \frac{\partial L(\mathbf{\Theta}_{\text{all}}; \mathbf{X}, \mathbf{Y})}{\partial \mathbf{\Theta_{xy}}} \\ \frac{\partial L(\mathbf{\Theta}_{\text{all}}; \mathbf{X}, \mathbf{Y})}{\partial \mathbf{\Theta_{yy}}} \end{pmatrix}, \tag{1}$$

where

$$\begin{aligned} \frac{\partial L(\mathbf{\Theta}_{\text{all}}; \mathbf{X}, \mathbf{Y})}{\partial \mathbf{\Theta_{xy}}} &= \mathbf{X}^T\mathbf{Y} - \sum_i E\big[\mathbf{x}^i {\mathbf{y}^i}^T\big], \\ \frac{\partial L(\mathbf{\Theta}_{\text{all}}; \mathbf{X}, \mathbf{Y})}{\partial \mathbf{\Theta_{yy}}} &= \frac{1}{2}\mathbf{Y}^T\mathbf{Y} - \frac{1}{2}\sum_i E\big[\mathbf{y}^i {\mathbf{y}^i}^T\big]. \end{aligned}$$

The expectations in the above equations are taken with respect to $p(\mathbf{y}^i|\mathbf{x}^i, \mathbf{\Theta_{xy}}, \mathbf{\Theta_{yy}})$ and can be com-

Algorithm 1 Optimization Algorithm for Sparse CGGM

Input: $\mathbf{X}$, $\mathbf{Y}$, $\mathbf{\Theta}^0_{\text{all}}$, line search parameters $l_0 > 0$ and $\alpha \in (0, 1)$.
Initialization: $\delta_0 = 1$, $\mathbf{U}^0 = \mathbf{\Theta}^0_{\text{all}}$, $l = l_0$.
Iterate: For iteration $t = 0, 1, 2, \ldots$, until convergence

1. Update $\delta_{t+1} = \frac{\sqrt{\delta_t^4 + 4\delta_t^2} - \delta_t^2}{2}$.

2. Update $\mathbf{W} = (1 - \delta_t)\mathbf{\Theta}^t_{\text{all}} + \delta_t \mathbf{U}^t$.

3. Compute $L(\mathbf{W}; \mathbf{X}, \mathbf{Y})$, $\nabla L(\mathbf{W}; \mathbf{X}, \mathbf{Y})$ according to Eq. (1).

4. Line search: Find the smallest non-negative integer $i \geq 0$ for $\bar{l} = l/\alpha^i$, such that $\overline{\mathbf{\Theta}}_{\text{all}}$ and $\overline{\mathbf{U}}$ are positive definite and

$$L(\overline{\mathbf{\Theta}}_{\text{all}}; \mathbf{X}, \mathbf{Y}) \leq L(\mathbf{W}; \mathbf{X}, \mathbf{Y}) + < \nabla L(\mathbf{W}; \mathbf{X}, \mathbf{Y}), \overline{\mathbf{\Theta}}_{\text{all}} - \mathbf{W} > + \frac{\bar{l}}{2} ||\overline{\mathbf{\Theta}}_{\text{all}} - \mathbf{W}||_2^2, \quad (2)$$

where

$$\overline{\mathbf{\Theta}}_{\text{all}} = \mathcal{T}_{\left[\frac{\lambda_1}{\bar{l}}, \frac{\lambda_2}{\bar{l}}\right]}\Big(\mathbf{W} - \frac{1}{\bar{l}}\nabla f(\mathbf{W})\Big), \quad \overline{\mathbf{U}} = \mathcal{T}_{\left[\frac{\lambda_1}{\delta_t \bar{l}}, \frac{\lambda_2}{\delta_t \bar{l}}\right]}\Big(\mathbf{U}^t - \frac{1}{\delta_t \bar{l}}\nabla f(\mathbf{W})\Big)$$

with $\mathcal{T}_{[a,b]}(\mathbf{D})$ defined as in Eq. (3).

5. Set $l = \bar{l}$, $\mathbf{\Theta}^{t+1}_{\text{all}} = \overline{\mathbf{\Theta}}_{\text{all}} = \mathcal{T}_{\left[\frac{\lambda_1}{l}, \frac{\lambda_2}{l}\right]}\Big(\mathbf{W} - \frac{1}{l}\nabla f(\mathbf{W})\Big)$, $\mathbf{U}^{t+1} = \overline{\mathbf{U}} = \mathcal{T}_{\left[\frac{\lambda_1}{\delta_t l}, \frac{\lambda_2}{\delta_t l}\right]}\Big(\mathbf{U}^t - \frac{1}{\delta_t l}\nabla f(\mathbf{W})\Big)$.

Output: $\hat{\mathbf{\Theta}}_{\text{all}} = \mathbf{\Theta}^{t+1}_{\text{all}}$.

---

puted as

$$\begin{aligned}
E\big[\mathbf{x}^i \mathbf{y}^{i^T}\big] &= -\mathbf{x}^i \mathbf{x}^{i^T} \mathbf{\Theta}_{\mathbf{xy}} \mathbf{\Theta}_{\mathbf{yy}}^{-1}, \\
E\big[\mathbf{y}^i \mathbf{y}^{i^T}\big] &= \text{Cov}(\mathbf{y}^i) + E\big[\mathbf{y}^i\big]E\big[\mathbf{y}^i\big]^T \\
&= \mathbf{\Theta}_{\mathbf{yy}}^{-1} + \mathbf{\Theta}_{\mathbf{yy}}^{-1} \mathbf{\Theta}_{\mathbf{xy}}^T \mathbf{x}^i \mathbf{x}^{i^T} \mathbf{\Theta}_{\mathbf{xy}} \mathbf{\Theta}_{\mathbf{yy}}^{-1}.
\end{aligned}$$

Steps 4 and 5 of Algorithm 1 are the key steps of the algorithm and perform the proximal gradient step. Step 4 performs a line search to obtain the appropriate gradient-descent step size by iteratively increasing the gradient step size parameter $\bar{l}$ and checking line-search stopping conditions in each iteration. The stopping condition in Eq. (2) ensures that the local quadratic approximation of the negative data log-likelihood on the right-hand side of the inequality is an upper-bound of the negative data log-likelihood on the left-hand side of the inequality. Given the gradient step size obtained from Step 4, Step 5 performs a proximal gradient step, where a gradient update $\mathbf{W}' = \mathbf{W} - \frac{1}{l}\nabla L(\mathbf{W}; \mathbf{X}, \mathbf{Y})$ is first performed with respect to the direction $\nabla L(\mathbf{W}; \mathbf{X}, \mathbf{Y})$ and then, the proximal mapping for the non-smooth L_1 penalty is performed as a soft-thresholding operation given as

$$\mathcal{T}_{\left[\frac{\lambda_1}{l}, \frac{\lambda_2}{l}\right]}\Big(\mathbf{W}'\Big),$$

where

$$\mathcal{T}_{\left[\frac{\lambda_1}{l}, \frac{\lambda_2}{l}\right]}(\mathbf{W}'_{(j,k)}) = \text{sign}(\mathbf{W}'_{(j,k)}) \max(0, |\mathbf{W}'_{(j,k)}| - v_{j,k}), \quad (3)$$

with $v_{j,k} = \frac{\lambda_1}{l}$ if $\mathbf{W}'_{(j,k)}$ is an element of $\mathbf{W}'_{\mathbf{xy}}$ and $v_{j,k} = \frac{\lambda_2}{l}$ if $\mathbf{W}'_{(j,k)}$ is an element of $\mathbf{W}'_{\mathbf{yy}}$. $\mathbf{U}$ is updated in a similar manner to $\mathbf{W}$.

The per-iteration computational time complexity of Algorithm 1 is $O(K^3 + J^2K + JK^2)$. The main computational cost arises from the inversion of the $K \times K$ matrix $\boldsymbol{\Theta}_{\mathbf{yy}}$ with the cost of $O(K^3)$ that is necessary for computing the gradient of objective with respect to $\boldsymbol{\Theta}_{\mathbf{yy}}$.
