# Supplementary material for: Correction: Learning Gene Networks under SNP Perturbations Using eQTL Datasets
Source: PLoS Comput Biol. 2014 Apr 9;10(4):e1003608. doi: 10.1371/journal.pcbi.1003608 (PMC3981648; doi:10.1371/journal.pcbi.1003608)
Supplement: File S1 — Originally published, uncorrected article. (PDF) [file pcbi.1003608.s001.pdf]

OPEN ACCESS Freely available online

PLOS | COMPUTATIONAL BIOLOGY

Learning Gene Networks under SNP Perturbations Using eQTL Datasets

Lingxue Zhang, Seyoung Kim*

Lane Center for Computational Biology, School of Computer Science, Carnegie Mellon University, Pittsburgh, Pennsylvania, United States of America

Abstract

The standard approach for identifying gene networks is based on experimental perturbations of gene regulatory systems such as gene knock-out experiments, followed by a genome-wide profiling of differential gene expressions. However, this approach is significantly limited in that it is not possible to perturb more than one or two genes simultaneously to discover complex gene interactions or to distinguish between direct and indirect downstream regulations of the differentially-expressed genes. As an alternative, genetical genomics study has been proposed to treat naturally-occurring genetic variants as potential perturbants of gene regulatory system and to recover gene networks via analysis of population gene-expression and genotype data. Despite many advantages of genetical genomics data analysis, the computational challenge that the effects of multifactorial genetic perturbations should be decoded simultaneously from data has prevented a widespread application of genetical genomics analysis. In this article, we propose a statistical framework for learning gene networks that overcomes the limitations of experimental perturbation methods and addresses the challenges of genetical genomics analysis. We introduce a new statistical model, called a sparse conditional Gaussian graphical model, and describe an efficient learning algorithm that simultaneously decodes the perturbations of gene regulatory system by a large number of SNPs to identify a gene network along with expression quantitative trait loci (eQTLs) that perturb this network. While our statistical model captures direct genetic perturbations of gene network, by performing inference on the probabilistic graphical model, we obtain detailed characterizations of how the direct SNP perturbation effects propagate through the gene network to perturb other genes indirectly. We demonstrate our statistical method using HapMap-simulated and yeast eQTL datasets. In particular, the yeast gene network identified computationally by our method under SNP perturbations is well supported by the results from experimental perturbation studies related to DNA replication stress response.

Citation: Zhang L, Kim S (2014) Learning Gene Networks under SNP Perturbations Using eQTL Datasets. PLoS Comput Biol 10(2): e1003420. doi:10.1371/journal.pcbi.1003420

Editor: Kevin Chen, Rutgers University, United States of America

Received July 23, 2013; **Accepted** November 18, 2013; **Published** February 27, 2014

Copyright: © 2014 Zhang, Kim. This is an open-access article distributed under the terms of the Creative Commons Attribution License, which permits unrestricted use, distribution, and reproduction in any medium, provided the original author and source are credited.

Funding: This material is based upon work supported by an NSF CAREER Award under grant No. MCB-1149885, Sloan Research Fellowship, and Okawa Foundation Research Grant. The funders had no role in study design, data collection and analysis, decision to publish, or preparation of the manuscript.

Competing Interests: The authors have declared that no competing interests exist.

* E-mail: sssykim@cs.cmu.edu

Introduction

Recent advances in the next-generation sequencing and other high-throughput technology has allowed researchers to collect various types of genome-scale datasets, providing unprecedented opportunities to discover detailed gene regulation processes in cells via analysis of the massive data. The standard approaches for identifying the causal regulatory relationship among genes have been based on examining gene-expression data collected after an experimental perturbation of one or two genes such as in gene knockout studies [1,2] or over-expression studies [3]. In a typical experimental design, genome-wide gene-expression levels are measured using microarrays under different experimental conditions such as strains with one or two genes knocked out [1,2]. Then, the differential gene-expression patterns between control and experimental conditions are examined to obtain clues as to the organization of genes into functional modules and key regulators of those modules.

However, this standard approach for identifying the wiring of gene networks based on experimental perturbations of gene regulation system comes with many limitations. Experimentally perturbing the activity of a gene can be very costly, time-consuming, and laborious and it is even more so for repeating such perturbation for every single gene in an organism to obtain a comprehensive picture of gene network wiring. Furthermore, the experimental methods are usually limited to a perturbation of one or two genes at a time due to experimental infeasibility and combinatorial explosion in the number of experiments to perform. Thus, they cannot be used to perturb more than two genes at the same time to obtain information on multifactorial gene interactions. More importantly, it is often not possible to apply such experimental perturbation studies to humans for ethical reasons. Finally, given the set of differentially expressed genes under each perturbation, it is difficult to distinguish between those genes that are directly regulated by the perturbed gene and those genes in the downstream of the pathway whose expressions are influenced as secondary/indirect effects.

Genetical genomics approach has been proposed as a less expensive but more powerful alternative to the approach with experimental perturbations [4,5]. Genetical genomics treats genetic variation as naturally-occurring perturbation of gene regulatory networks and tries to learn gene networks by examining the effects of genetic variation on gene expression measurements within a large population of individuals. The key advantage of genetical genomics approach is that unlike experimental perturbations that can be performed only on one or two genes at a time,

Author Summary

A complete understanding of how gene regulatory networks are wired in a biological system is important in many areas of biology and medicine. The most popular method for investigating a gene network has been based on experimental perturbation studies, where the expression of a gene is experimentally manipulated to observe how this perturbation affects the expressions of other genes. Such experimental methods are costly, laborious, and do not scale to a perturbation of more than two genes at a time. As an alternative, genetical genomics approach uses genetic variants as naturally-occurring perturbations of gene regulatory system and learns gene networks by decoding the perturbation effects by genetic variants, given population gene-expression and genotype data. However, since there exist millions of genetic variants in genomes that simultaneously perturb a gene network, it is not obvious how to decode the effects of such multifactorial perturbations from data. Our statistical approach overcomes this computational challenge and recovers gene networks under SNP perturbations using probabilistic graphical models. As population gene-expression and genotype datasets are routinely collected to study genetic architectures of complex diseases and phenotypes, our approach can directly leverage these existing datasets to provide a more effective way of identifying gene networks.

there are more than millions of genetic variants across genomes in the case of a human population, enabling the effects of multifactorial perturbations to be observed directly in gene expression data. Another advantage is that while experimental perturbation studies involve artificial perturbations in lab with often large perturbation effects, the perturbations of gene network by genetic variants occur in nature and usually induce more subtle changes in gene expressions. Thus, understanding the consequences of network perturbations by genetic variants is likely to lead to more direct understanding of the gene networks that exist in nature. In addition, genetical genomics approach can be easily applied to humans as well as other organisms, since genotype and gene-expression data are routinely collected for the purpose of expression quantitative trait locus (eQTL) mapping to understand the genetic architecture of complex phenotypes and diseases [6,7].

However, the genetical genomics approach poses a significant computational challenge, because it is not obvious how to decode the effects of multifactorial genetic perturbations from genotype and gene expression data. In an experimental approach with a perturbation of one or two genes, the genes that are differentially expressed under each perturbation experiment can be easily identified with a simple computation. However, in genetical genomics approach, the gene expression variability is the result of aggregated effects of multifactorial perturbations by a large number of genetic variants and it is not obvious how to decouple the aggregated perturbation effects to identify the set of genes that are differentially expressed with respect to each perturbation by each individual genetic variants. Furthermore, as many of the genetic variants do not have any functional consequences or perturbation effects, genetical genomics approach has an additional computational challenge of identifying eQTLs or the genetic variants that affect gene expressions, while identifying the gene network perturbed by these eQTLs at the same time. Because of these computational challenges, given gene-expression and genotype data, researchers tended to limit their analysis to eQTL mapping, where eQTL mapping can be viewed as a special case of genetical genomics analysis that assumes an isolated effect of genetic perturbation on a single gene with no downstream effects in gene network. While statistical methods such as graph-guided fused lasso (GFlasso) [8] have been developed to detect genetic effects on multiple correlated gene-expression traits, they focused only on eQTL mapping assuming a known gene network, instead of performing a more powerful genetical genomics analysis. Those few existing computational methods for genetical genomics analysis have been limited in terms of computational efficiency and statistical power [9–12]. For example, discovering eQTLs and reconstructing the gene network perturbed by those eQTLs were performed in two separate steps, leading to reduced statistical power [9], or average gene-expression levels within each gene module were used as a trait to identify eQTLs rather than using the original gene-expression data, leading to the loss of information on individual gene activities [13].

In this paper, we propose a statistical framework that directly addresses all of the above computational challenges of genetical genomics analysis within a single statistical analysis to achieve the maximum statistical power for identifying gene networks via single-nucleotide-polymorphism (SNP) perturbations. Given SNP genotype and gene-expression data collected for a large number of individuals, our statistical method simultaneously identifies 1) the gene network structure by decoding the effects of multifactorial perturbations of gene regulation system by a large number of SNPs, 2) eQTLs that perturb this gene network, 3) the genes whose expressions are directly perturbed by eQTLs and the genes whose expressions are indirectly perturbed as secondary downstream effects of the direct perturbations in the network, and 4) detailed characterizations of the SNP perturbation effects on the gene network by decoupling the complex multifactorial SNP effects on the gene network with respect to each individual perturbation.

Our proposed statistical framework is based on probabilistic graphical models, and in particular, we introduce a new statistical model, called a sparse conditional Gaussian graphical model (CGGM), that models a gene network under SNP perturbations as an undirected graphical model. In our statistical model, the unknown gene network is represented as a graph over gene-expression traits and this graph is associated with an unknown probability distribution that models the strengths of gene-gene interactions in the gene network and the strengths of perturbation effects of SNPs (Figure 1A). Then, both the gene network structure perturbed by SNPs and probability distribution associated with the network structure are learned jointly from data. We show that the learning problem is convex, leading to increased statistical power and a guarantee in the quality of the estimated model, and develop an efficient learning algorithm that scales to a large dataset. Given the estimated graphical model, we describe inference methods to characterize the detailed mechanisms of how the effects of SNP perturbations propagate through the network (Figures 1B–D).

From the computational point of view, addressing the challenges of genetical genomics analysis requires handling the computational challenges of both gene-network analysis given gene-expression data and eQTL mapping given gene-expression and genotype data, namely gene-network structure learning and SNP feature selection, at the same time. We show that in fact our sparse CGGM subsumes as special cases both a sparse Gaussian graphical model [14–17], which is popular as a model for gene network, and a sparse linear regression model [8,18,19], which is widely used for eQTL mapping, thus providing a natural unifying representation for a gene network and eQTLs perturbing this network. Moreover, by embedding the standard regression model for eQTL mapping within a probabilistic graphical model and leveraging the representational power of a graphical model, our

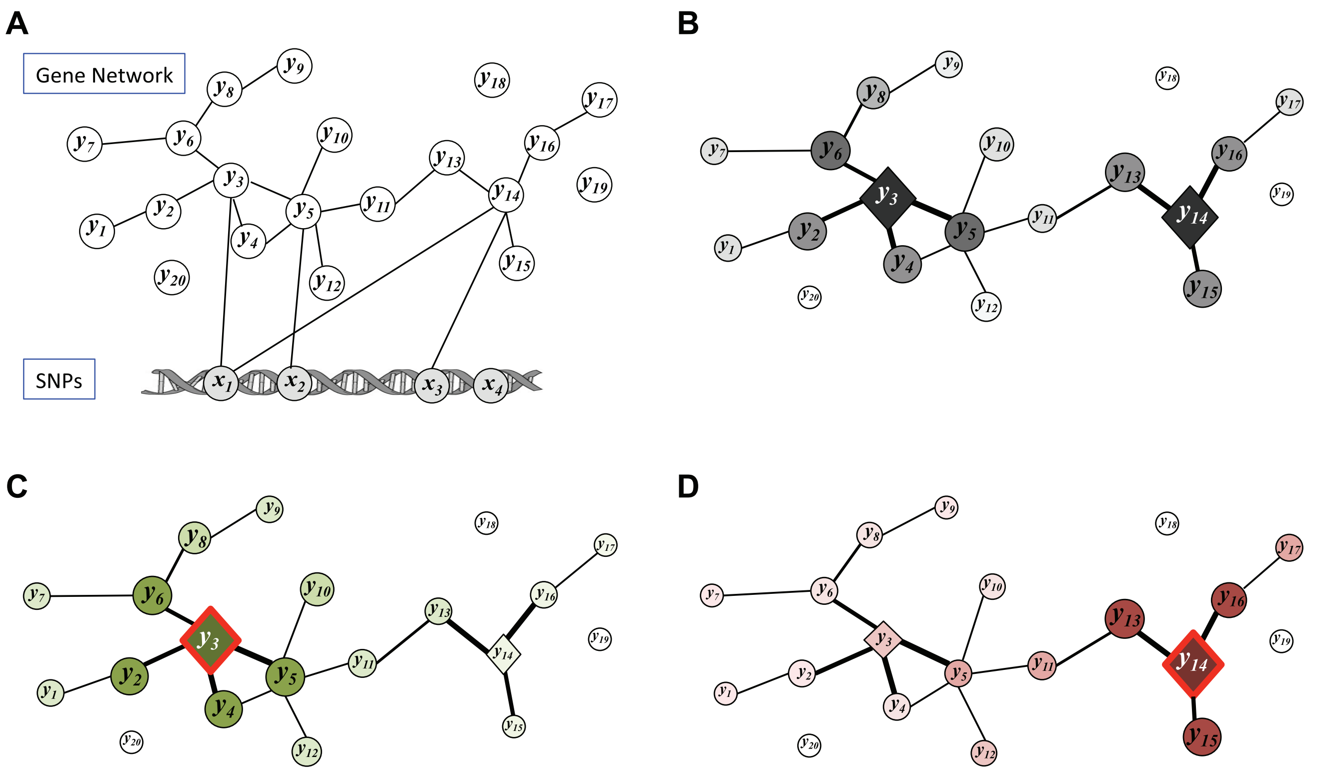

Figure 1. Illustration of our statistical framework for learning a gene network with genetical genomics analysis. (A) The graph structure of sparse CGGM for modeling a gene network perturbed by SNPs. The gene network is defined over gene-expression traits y_k's ($k=1,\ldots,20$). The edges between gene-expression traits y_k's and SNPs x_j's ($j=1,\ldots,4$) indicate the direct perturbations of the gene-expression traits by the given SNPs. The nodes for SNPs x_j's are shaded to show that the SNPs are conditioning variables in the conditional probability model. (B) Illustration of how the effects of the direct perturbation of the gene network by SNP x_1 propagate through the gene network, as obtained by performing inference on sparse CGGM in Panel (A). While SNP x_1 perturbs gene-expression traits y_3 and y_{14} directly, this effect propagates through the network to perturb the expressions of other genes indirectly. The two directly perturbed genes y_3 and y_{14} are shown as diamond-shaped nodes. The size and color-shade of each node indicate the strength of indirect perturbation of the given gene-expression trait by SNP x_1, with a larger and darker node for stronger perturbation. (C) The portion of the overall indirect SNP perturbation effects in Panel (B) that arose from the propagation of the direct perturbation of gene y_3 by SNP x_1. (D) The portion of the overall indirect SNP perturbation effects in Panel (B) that arose from the propagation of the direct perturbation of gene y_{14} by SNP x_1. Within our statistical framework, we can perform inference on sparse CGGM in Panel (A) to obtain the indirect perturbations in Panel (B), and then decompose the indirect perturbations in Panel (B) into Panels (C) and (D) in a principled manner.
doi:10.1371/journal.pcbi.1003420.g001

approach allows to extract a significantly more detailed characterization of the functional roles of eQTLs than any of the existing methods for eQTL mapping.

In our experiments, we apply our statistical framework to HapMap-simulated and yeast eQTL datasets [20,21]. Using HapMap-simulated data, we demonstrate our approach can recover the true underlying gene network under SNP perturbations, and at the same time, can recover true eQTLs with greater statistical power than other existing methods that have been developed for eQTL mapping. In addition, we applied our method to yeast eQTL dataset collected for 112 segregants of two yeast parent strains, BY4716 and RM11-1a [21]. Nearly all of the previous analyses of this dataset focused either on eQTL mapping or on gene network analysis ignoring the genetic information, with an exception of the genetical genomics analysis of this dataset performed by multivariate regression with covariance estimation (MRCE) [22] that has also been mistakenly called sparse CGGM [23] as we further discuss in the next section. However, the analysis by MRCE [23] was performed for only a small subset of the full dataset because of its expensive computational cost. In our experiment, MRCE took more than weeks of computation to analyze the full yeast eQTL dataset, whereas our method ran within a day. We provide an in-depth analysis of SNP perturbations of a subnetwork over genes involved in stress response in yeast, and show that this subnetwork obtained from SNP perturbations by our approach is well supported by the network inferred from experimental perturbations in knock-out studies in the literature.

Materials and Methods

We begin our discussion with a brief background on sparse Gaussian graphical models for learning gene networks from gene expression data and sparse regression models for identifying eQTLs from gene expression and SNP data. Then, we present our proposed statistical framework for genetical genomics analysis and describe the model, learning algorithm, and inference procedure for sparse CGGM.

Background on Sparse Gaussian Graphical Models for Gene Network Learning

A Gaussian graphical model defines a probability distribution over an undirected graph that models a gene network. The nodes of the graph correspond to continuous-valued random variables for gene-expression traits and the edges represent probabilistic conditional

dependence relationships between pairs of nodes [14–17]. Given microarray gene-expression measurements $\mathbf{Y}=[\mathbf{y}^1,\ldots,\mathbf{y}^N]^T\in\mathbb{R}^{N\times K}$ for K genes and N individuals, a Gaussian graphical model assumes that the gene-expression measurement $\mathbf{y}^i=[y^i_{(1)},\ldots,y^i_{(K)}]^T$ for the ith individual is an independently and identically distributed sample from a Gaussian distribution $N(0_K,\mathbf{\Sigma})$, where 0_K is a vector of K 0's and $\mathbf{\Sigma}$ is a $K\times K$ covariance matrix. Then, it is well-known that the inverse covariance $\mathbf{\Theta}=\mathbf{\Sigma}^{-1}$ represents a Gaussian graphical model, where the non-zero (or zero) value for $\theta_{(k,l)}$ in the (k,l)th entry of $\mathbf{\Theta}$ represents the presence (or absence) of edges between the kth and lth gene-expression traits in the gene network. While each non-zero element $\theta_{(k,l)}$ in $\mathbf{\Theta}$ implies conditional dependency between the kth and lth gene-expression traits given all the other gene-expression traits, computing the inverse of $\mathbf{\Theta}$ to obtain the covariance $\mathbf{\Sigma}=\mathbf{\Theta}^{-1}$ amounts to performing an inference in this graphical model to obtain marginal dependencies, or equivalently dependencies between the two nodes without consideration of any other nodes.

Graphical lasso [14,16,24] has been widely used to learn a sparse Gaussian graphical model, where only statistically significant gene-gene interactions have edges with non-zero entries in $\mathbf{\Theta}$. Graphical lasso minimizes the negative log-likelihood with sparsity-inducing L_1 penalty as follows:

$$\arg\min_{\mathbf{\Theta}} \quad -\log\det\mathbf{\Theta}+\mathrm{tr}(\mathbf{S}\,\mathbf{\Theta})+\lambda\|\mathbf{\Theta}\|_1, \qquad (1)$$

where $\mathrm{tr}(\mathbf{A})$ is the trace of matrix $\mathbf{A}$, $\mathbf{S}$ is the sample covariance matrix, $\|\mathbf{\Theta}\|_1=\sum_k\sum_l\left|\theta_{(k,l)}\right|$ is the L_1 norm of $\mathbf{\Theta}$, and λ is the regularization parameter that determines the amount of sparsity. A large value of λ leads to a sparser estimate with a greater number of zero elements in $\mathbf{\Theta}$. The optimal value for λ can be determined using cross-validation. The problem in Eq. (1) is convex and can be solved efficiently [16].

Background on Sparse Regression Methods for eQTL Mapping

In eQTL mapping, the problem of identifying SNPs influencing gene-expression levels from eQTL data is often formulated as that of learning a multivariate linear regression model [8,18,19]. Given genotype data for J SNPs $\mathbf{X}=[\mathbf{x}^1,\ldots,\mathbf{x}^N]^T$, where $\mathbf{x}^i=[x^i_{(1)},\ldots,x^i_{(J)}]^T$ is a vector of length J with each element taking values from $\{0,1,2\}$ for the number of minor alleles at the given locus, and the expression measurements for K genes $\mathbf{Y}=[\mathbf{y}^1,\ldots,\mathbf{y}^N]^T\in\mathbb{R}^{N\times K}$ for N samples, a linear regression model for the functional mapping from SNPs to gene-expression traits is given as:

$$\mathbf{Y}=\mathbf{XB}+\mathbf{E}, \qquad (2)$$

where $\mathbf{B}\in\mathbb{R}^{J\times K}$ is the regression coefficient matrix representing the unknown association strengths, and $\mathbf{E}\in\mathbb{R}^{N\times K}$ is the matrix of noise terms whose rows are Gaussian-distributed with mean zeros and covariance $\mathbf{\Psi}$. Typically, a model without an intercept is considered, assuming that the genotype data are standardized to have mean 0 and unit variance.

Since each gene-expression trait typically has only a small number of eQTLs, lasso [19,25] has been widely used to obtain a sparse estimate of $\mathbf{B}$. Lasso minimizes the squared-error criterion with L_1 penalty as follows:

$$\arg\min_{\mathbf{B}} \ \mathrm{tr}((\mathbf{Y}-\mathbf{XB})(\mathbf{Y}-\mathbf{XB})^T)+\lambda\|\mathbf{B}\|_1, \qquad (3)$$

where λ is a regularization parameter that controls the amount of sparsity in $\mathbf{B}$. Eq. (3) is convex with a single globally optimal solution, and efficient algorithms are available for solving it [26].

Lasso essentially performs K separate regression analyses, treating the K gene-expression traits as independent of each other. In order to combine the statistical power across multiple correlated gene-expression traits, GFlasso [8] assumed a known gene network and extended the standard lasso by including an additional penalty, called graph-guided fusion penalty, that encourages multiple related genes in the gene network to be influenced pleiotropically by a common SNP. Given a gene network with a set of edges E and edge weights r_{mk}'s for each edge $(m,k)\in E$ between the mth and kth genes, the graph-guided fusion penalty takes the form of $J(\mathbf{B})=\sum_j\sum_{(m,k)\in E}|r_{mk}||\beta_{(j,m)}-\mathrm{sign}(r_{mk})\beta_{(j,k)}|$, where each term in the penalty encourages the amount of influence of the jth SNP on the expression levels of the mth and kth genes to be similar if the two genes are connected with an edge in the network. GFlasso was capable of identifying SNPs with pleiotropic effects, but it was restrictive in that the gene network should be known *a priori*.

Within the same statistical framework of linear regression method, MRCE [22] attempted to shift the focus from eQTL mapping towards genetical genomics analysis by identifying the gene network and eQTLs jointly. Towards this goal, MRCE relaxed the assumption of uncorrelated noise (i.e., $\mathbf{\Psi}$ being a diagonal matrix) in lasso and estimated the full noise covariance matrix $\mathbf{\Psi}$. Then, the inverse of the noise covariance $\mathbf{\Omega}=\mathbf{\Psi}^{-1}$ corresponds to a gene network. MRCE minimizes the negative log-likelihood of data with an L_1 penalty for both $\mathbf{B}$ and $\mathbf{\Omega}=\mathbf{\Psi}^{-1}$:

$$\begin{aligned}\arg\min_{\mathbf{B},\mathbf{\Omega}} \ & -N\log|\mathbf{\Omega}|+\mathrm{tr}((\mathbf{Y}-\mathbf{XB})\mathbf{\Omega}(\mathbf{Y}-\mathbf{XB})^T)\\ & +\lambda_1||\mathbf{B}||_1+\lambda_2||\mathbf{\Omega}||_1,\end{aligned} \qquad (4)$$

where λ_1 and λ_2 are the regularization parameters. We notice that unlike GFlasso, MRCE does not have any mechanisms to leverage the estimated gene network $\mathbf{\Omega}$ to model pleiotropic effects of SNPs on multiple correlated gene-expression traits in $\mathbf{B}$. The optimization problem in Eq. (4) is not convex, but bi-convex, since fixing either $\mathbf{B}$ or $\mathbf{\Omega}$ and solving for the other is a convex optimization problem. Thus, Rothman et al. [22] proposed to optimize for each of $\mathbf{B}$ and $\mathbf{\Omega}$ alternately given the other over iterations. However, they noted that this strategy often does not converge, and instead suggested to use an approximate method that prematurely terminates the iterative optimization procedure after two iterations. As we discuss in the Results section, we found that even this approximate method was too slow to be applicable to a dataset of even moderate size.

The same statistical method for MRCE has been proposed independently in the literature under the name of sparse conditional Gaussian graphical models (CGGMs) [23]. However, we emphasize that the statistical model that is learned in MRCE is not a graphical model, because as we further discuss in detail in the next sections, the parameters $\mathbf{B}$ do not model conditional dependencies as in graphical models but only models marginal dependencies. We believe that MRCE was mistakenly called a sparse CGGM due to the resemblance between the inverse noise covariance matrix $\mathbf{\Omega}$ in MRCE and the inverse covariance matrix $\mathbf{\Theta}$ in graphical lasso as well as the aspect of the standard regression model as a conditional model for $\mathbf{Y}$ given $\mathbf{X}$. The sparse CGGM that we propose in this paper is set up as a proper probabilistic graphical model and as we show in the next sections, is significantly more powerful than MRCE in terms of representational power and computational efficiency.

Under the same name of sparse CGGMs, Li et al. [27] introduced a related but different statistical method that also models a gene network corresponding to $\mathbf{\Omega}=\mathbf{\Psi}^{-1}$ in MRCE. However, unlike our approach and MRCE, the estimation procedure for the sparse CGGM in [27] amounted to a two-stage process, where the gene-expression data are pre-processed to remove SNP effects in the first stage and then these pre-processed gene-expression data are used to learn a gene network in the second stage. Thus, their graphical model was defined only on gene-expression traits and did not directly model the relationship between SNPs and gene expressions to identify eQTLs. In contrast, our sparse CGGM is set up as a graphical model on both gene expressions and SNPs, performs a joint estimation of gene network and eQTLs, and infers various perturbation effects of SNPs on gene expressions via inference.

CGGMs for Modeling Gene Networks under SNP Perturbations

In this section, we introduce a statistical model for CGGM as a model for a gene network under SNP perturbations. Then, in the next sections, we describe a learning algorithm for estimating a sparse model for CGGM from data and discuss inference schemes for the estimated sparse CGGM. A sparse CGGM estimated from data captures the gene network structure and direct perturbations of the gene-expression levels by eQTLs via conditional dependency structure in the estimated graph structure. By performing inference on this estimated sparse CGGM, we can obtain a detailed characterization of how the direct SNP perturbation effects propagate through the gene network to perturb the expression levels of other genes indirectly.

The key idea behind our proposed approach is to model a gene network under SNP perturbations as a Gaussian graphical model for a gene network conditional on SNPs. We derive a CGGM as a conditional distribution $p(\mathbf{y}^i|\mathbf{x}^i)$ from the Gaussian graphical model for a joint probability distribution $p(\mathbf{y}^i,\mathbf{x}^i)$ for SNPs $\mathbf{x}^i$ and gene-expression traits $\mathbf{y}^i$ for the ith individual. Let us assume a Gaussian graphical model $p(\mathbf{x}^i,\mathbf{y}^i)=N(0_{J+K},\mathbf{\Sigma})$ with covariance $\mathbf{\Sigma}=\begin{pmatrix}\mathbf{\Sigma_{xx}} & \mathbf{\Sigma_{xy}}\\ \mathbf{\Sigma_{xy}^{\mathit{T}}} & \mathbf{\Sigma_{yy}}\end{pmatrix}$ and inverse covariance $\mathbf{\Theta}=\mathbf{\Sigma}^{-1}=\begin{pmatrix}\mathbf{\Theta_{xx}} & \mathbf{\Theta_{xy}}\\ \mathbf{\Theta_{xy}^{\mathit{T}}} & \mathbf{\Theta_{yy}}\end{pmatrix}$, where zero mean is assumed after mean-centering each gene-expression trait and SNP. Then, the conditional distribution of $\mathbf{y}^i$ given $\mathbf{x}^i$ can be obtained as $p(\mathbf{y}^i|\mathbf{x}^i)=N(\mathbf{\Sigma_{xy}^{\mathit{T}}}\mathbf{\Sigma_{xx}^{-1}}\mathbf{x}^i,\ \mathbf{\Sigma_{yy}}-\mathbf{\Sigma_{xy}^{\mathit{T}}}\mathbf{\Sigma_{xx}^{-1}}\mathbf{\Sigma_{xy}})$. We further re-write this conditional distribution, using the inverse covariance matrix $\mathbf{\Theta}$ and the partitioned inverse formula [28] to obtain a CGGM:

$$p(\mathbf{y}^i|\mathbf{x}^i)=N(-\mathbf{\Theta_{yy}^{-1}}\mathbf{\Theta_{xy}^{\mathit{T}}}\mathbf{x}^i,\ \mathbf{\Theta_{yy}^{-1}}). \tag{5}$$

CGGM parameters $\mathbf{\Theta_{yy}}$ and $\mathbf{\Theta_{xy}}$ represent a gene network and SNP perturbation effects on this gene network, respectively. A non-zero value for the (j,k)th element of $\mathbf{\Theta_{xy}}$ indicates that the jth SNP is an eQTL for the kth gene-expression trait. This SNP perturbation captures the direct influence of the jth SNP on the kth gene expression, since the graphical model captures conditional dependencies. While in experimental perturbation studies the expressions of only one or two genes can be directly perturbed (e.g., by knocking out the genes), our CGGM for genetical genomics study allows multiple gene-expression traits to be perturbed by multiple SNPs at the same time. Then, this multifactorial genetic perturbations of gene-expression levels are decoded to learn a gene network by a learning algorithm that estimates $\mathbf{\Theta_{yy}}$ and $\mathbf{\Theta_{xy}}$ simultaneously.

In order to show the direct correspondence between a CGGM and a general undirected graphical model, we re-write Eq. (5) by expanding the quadratic term in the Gaussian distribution in Eq. (5) to obtain:

$$\begin{aligned}p(\mathbf{y}^i|\mathbf{x}^i)&=\exp(-1/2\,\mathbf{y}^i{}^T\mathbf{\Theta_{yy}}\mathbf{y}^i-\mathbf{x}^i{}^T\mathbf{\Theta_{xy}}\mathbf{y}^i)/Z,\\ &=\exp(-1/2\sum_{m,l}\theta_{yy(m,l)}y^i_{(m)}y^i_{(l)}-\sum_{k,j}\theta_{xy(j,k)}x^i_{(j)}y^i_{(k)})/Z,\end{aligned}\tag{6}$$

where $Z=\int\exp(-\frac{1}{2}\mathbf{y}^i{}^T\mathbf{\Theta_{yy}}\mathbf{y}^i-\mathbf{x}^i{}^T\mathbf{\Theta_{xy}}\mathbf{y}^i)d\mathbf{y}^i=\sqrt{\frac{(2\pi)^K}{\det\mathbf{\Theta_{yy}}}}\exp(\frac{1}{2}\mathbf{x}^i{}^T\mathbf{\Theta_{xy}}\mathbf{\Theta_{yy}^{-1}}\mathbf{\Theta_{xy}^{\mathit{T}}}\mathbf{x}^i)$ is a constant, also known as a partition function in the literature of probabilistic graphical models [29], which ensures that $p(\mathbf{y}^i|\mathbf{x}^i)$ forms a proper probability distribution integrating to 1. As Eq. (6) is equivalent to the Gaussian-distribution form in Eq. (5), this constant can be obtained in a closed-form by directly comparing Eq. (6) with Eq. (5). If $\mathbf{\Theta_{yy}}$ is positive definite, the integral in the partition function is finite and the probability distribution is well-defined. The representation in Eq. (6) explicitly shows that a CGGM is an undirected graphical model [29] defined over a graph with two sets of edges, namely the set of edges connecting each pair of gene-expression traits in gene network and another set of edges connecting each SNP to gene expressions that the SNP is influencing (Figure 1A). Then, following the definition of an undirected graphical model [29], the numerator in Eq. (6) is a weighted sum of features over the graph edges, where $y^i_{(m)}y^i_{(l)}$'s and $x^i_{(j)}y^i_{(k)}$'s are features and $\mathbf{\Theta_{yy}}$ and $\mathbf{\Theta_{xy}}$ define edge weights. The gene network is modeled an undirected graph, but the directions from SNPs to gene-expression traits are implicit, since the model is a conditional probability model for gene-expression traits conditional on SNPs.

Learning Sparse CGGMs

Since both gene-gene interactions and SNP perturbations of gene-expression traits are highly modular and localized, we are interested in learning a sparse model for CGGM. In other words, only statistically significant gene-gene interactions should be represented as edges with non-zero entries in $\mathbf{\Theta_{yy}}$ and only a small number of statistically significant direct SNP perturbations should be estimated as having non-zero effect sizes in $\mathbf{\Theta_{xy}}$. In order to impose a sparsity constraint, we learn a sparse CGGM by minimizing the negative log-likelihood of data with an L_1 penalty as follows:

$$\arg\min_{\mathbf{\Theta_{yy}},\mathbf{\Theta_{xy}}} L(\mathbf{\Theta_{xy}},\mathbf{\Theta_{yy}};\mathbf{X},\mathbf{Y})+\lambda_1\|\mathbf{\Theta_{xy}}\|_1+\lambda_2\|\mathbf{\Theta_{yy}}\|_1, \tag{7}$$

where $L(\mathbf{\Theta_{xy}},\mathbf{\Theta_{yy}};\mathbf{X},\mathbf{Y})=1/2\,\mathrm{tr}(\mathbf{Y}\mathbf{\Theta_{yy}}\mathbf{Y}^T)+\mathrm{tr}(\mathbf{X}\mathbf{\Theta_{xy}}\mathbf{Y}^T)+\sum_i\log Z(\mathbf{\Theta_{xy}},\mathbf{\Theta_{yy}},\mathbf{x}^i)$ is the negative log-likelihood of data based on Eq. (6) or equivalently Eq. (5), and λ_1 and λ_2 are the regularization parameters that control the amount of sparsity. It is not necessary to explicitly consider the positive-definite constraint for $\mathbf{\Theta_{yy}}$ within the optimization problem in Eq. (7), because the partition function in the data log-likelihood contains $\log\det(\mathbf{\Theta_{yy}})$ term that acts as a log-barrier function for the positive-definite constraint [30]. Within the L_1 penalty for $\mathbf{\Theta_{yy}}$, we do not penalize the diagonal elements of $\mathbf{\Theta_{yy}}$, since we found that this leads to a slightly better performance in our experiments, consistent with what has been reported for graphical lasso. It is straightforward to prove that the

problem in Eq. (7) is convex (Text S1) [30]. Thus, the learning algorithm is guaranteed to find the globally optimal solution that achieves the maximum statistical power.

The main challenge for solving Eq. (7) arises from the non-smoothness of the L_1 penalty function. We adopt a variant of accelerated proximal gradient algorithms, called a Nesterov's second method [31], that has been developed as a general-purpose algorithm for handling a non-smooth component of the parameter estimation problem while improving the convergence (and thus, computation time) of the standard gradient descent algorithm [31–33]. We provide details of the learning algorithm in Text S2.

Lasso [25] and graphical lasso [14,16,24] can be viewed as special cases of the sparse CGGM estimation problem in Eq. (7). When $\lambda_1 >> \lambda_2$, the sparse CGGM learning problem in Eq. (7) essentially reduces to applying graphical lasso to gene-expression data, ignoring genotype data, since the large λ_1 encourages all or nearly all of the elements of $\mathbf{\Theta_{xy}}$ to be set to zeros. On the other hand, if $\lambda_1 << \lambda_2$, the sparse CGGM learning problem becomes equivalent to lasso that fits a regression model for each gene-expression trait separately, ignoring gene network, since the large λ_2 tends to set all or almost all of the off-diagonal elements of $\mathbf{\Theta_{yy}}$ to zeros. The optimal values for λ_1 and λ_2 that strike the right balance between these two extreme cases can be found by cross-validation.

Inference on Sparse CGGMs for Detailed Characterizations of SNP Perturbations of Gene Networks

So far, we showed that by learning a sparse CGGM, it is possible to decode the underlying gene network and its direct multifactorial perturbations by SNPs from data. Now, we show that given a sparse CGGM estimated from data, we can perform inference on this graphical model to characterize the mechanisms of SNP perturbations of gene network in detail. Below, we discuss how inference schemes can be used on our estimated model to learn about indirect/secondary downstream effects of the direct SNP perturbations, a decomposition of the overall multifactorial SNP perturbation effects with respect to each individual direct perturbation, and a decomposition of observed covariance in gene expressions into genetic and non-genetic components. We note that all of these inference schemes involve only few simple matrix operations and are highly efficient.

Direct and Indirect SNP Perturbations of Gene Network. Unlike experimental perturbation studies for learning a gene network, in genetical genomics analysis with our statistical framework, the properties of a sparse CGGM as a graphical model allow us to learn whether the differential gene expression arises from direct or indirect/secondary effects of the SNP perturbation. Direct perturbations of gene-expression traits by SNPs are encoded explicitly as conditional dependencies or as the sparsity pattern of $\mathbf{\Theta_{xy}}$ in the estimated sparse CGGM. Then, performing inference on this graphical model is equivalent to inferring indirect SNP perturbations of gene-expression traits, which arose from the effects of direct SNP perturbations propagating through the gene network to affect other neighboring gene-expression traits indirectly and pleiotropically.

More specifically, while Eq. (6) provides the standard form of a CGGM as a graphical model (Figure 1A), obtaining the form in Eq. (5) from Eq. (6) amounts to performing inference in the CGGM (Figure 1B). In other words, performing inference in a CGGM parameterized by $\mathbf{\Theta_{yy}}$ and $\mathbf{\Theta_{xy}}$ is equivalent to computing $\mathbf{B} = [-\mathbf{\Theta_{yy}^{-1}}\mathbf{\Theta_{xy}^{T}}]^T = -\mathbf{\Theta_{xy}}\mathbf{\Theta_{yy}^{-1}}$ and $\mathbf{\Psi} = \mathbf{\Theta_{yy}^{-1}}$, where the choice of notation $\mathbf{B}$ and $\mathbf{\Psi}$ was deliberate due to the connection between inference in a CGGM and the standard regression model in Eq. (2) as discussed in the next section. Thus, even if the jth SNP does not perturb the kth gene-expression trait (i.e., the (j,k)th entry of $\mathbf{\Theta_{xy}}$ is zero), if the (j,k)th entry of $\mathbf{B}$ is non-zero, the jth SNP perturbs the kth gene-expression trait indirectly, as a result of the downstream effects of the direct perturbation of other genes in the network by the jth SNP.

More generally, indirect SNP perturbations can be inferred by examining the sparsity pattern of $\mathbf{B}$. The indirect SNP perturbations as captured by the sparsity pattern of $\mathbf{B}$ in sparse CGGM is determined by both direct SNP perturbations (the sparsity pattern of $\mathbf{\Theta_{xy}}$) and the topology of the estimated gene network (the sparsity pattern of $\mathbf{\Theta_{yy}}$). For example, if the gene network $\mathbf{\Theta_{yy}}$ contains multiple connected components, where each connected component is a network over a subset of gene-expression traits without any edges across different connected components, the effects of direct SNP perturbations propagate to influence other gene-expression traits only within the given connected component. This leads to a sparsity pattern in $\mathbf{B}$, where a SNP directly influencing a gene-expression trait in $\mathbf{\Theta_{xy}}$ has non-zero indirect perturbation effects in $\mathbf{B}$ for all of the gene-expression traits within the same connected component. On the other hand, if $\mathbf{\Theta_{yy}}$ is a diagonal matrix with no edges in the gene network, the direct SNP perturbation effects do not propagate through the gene network, and the sparsity pattern in $\mathbf{B}$ remains the same as that of $\mathbf{\Theta_{xy}}$. More generally, gene network $\mathbf{\Theta_{yy}}$ plays the role of calibrating how the effects of direct genetic perturbations in $\mathbf{\Theta_{xy}}$ propagate to affect other gene-expression traits indirectly in $\mathbf{B}$, such that an eQTL has strong pleiotropic effects within strongly-connected parts of the gene network.

Decomposition of SNP Perturbation Effects. Given a sparse CGGM, the amount of the overall influence of a SNP on a particular gene-expression trait in $\mathbf{B}$ can be further decomposed into contributions from each of the directly-perturbed genes that pass the perturbation effects to other parts of the network. For example, while each row of $\mathbf{B}$ captures the overall direct/indirect perturbation effects of the given SNP on gene network (Figure 1B), this overall perturbation effects can be decomposed with respect to each of the genes that are directly perturbed by the same SNP (Figures 1C and D). In experimental perturbation studies, only one or two genes are perturbed, but in genetical genomics analysis with multifactorial perturbations, each SNP can directly perturb multiple genes at the same time, such as mutations in a transcription factor perturbing expressions of multiple target genes. We show that a simple matrix operation for a decomposition of $\mathbf{B}$ can identify how each of the directly perturbed genes propagate the perturbation effects to other genes.

More specifically, the overall indirect perturbation effects $\mathbf{B}$ can be decomposed into $\mathbf{B}_k$'s, $k = 1, \ldots, K$, as follows:

$$\begin{aligned}\mathbf{B} &= -\mathbf{\Theta_{xy}}\mathbf{\Theta_{yy}^{-1}} = -\mathbf{\Theta_{xy}}\mathbf{\Psi} \\ &= -\sum_{k=1}^{K}\mathbf{\Theta}_{\mathbf{xy}(:,k)}\mathbf{\Psi}_{(k,:)} = \sum_{k=1}^{K}\mathbf{B}_k,\end{aligned} \quad (8)$$

where $\mathbf{A}_{(:,z)}$ and $\mathbf{A}_{(z,:)}$ represent the zth column and row of matrix $\mathbf{A}$, respectively. $\mathbf{B}_k$ contains information on how the kth gene passes on the direct SNP perturbation effects it received to other gene-expression traits. Figures 1B, C, and D illustrate a single row of each of $\mathbf{B}$, $\mathbf{B}_3$, and $\mathbf{B}_{14}$, respectively, corresponding to SNP x_1 and gene-expression traits y_3 and y_{14} in the graphical model in Figure 1A.

Decomposition of Gene-Expression Covariance. In addition, given the estimate of sparse CGGM parameters, the overall

observed covariance in gene-expression data can be approximately decomposed into the covariance induced by the gene network $\mathbf{\Theta_{yy}}$ and the covariance induced by direct SNP perturbations propagating to different parts of the network. In order to derive this decomposition, ignoring the L_1 penalty that introduces a sparsity bias, we consider the problem of finding the optimal CGGM parameter $\mathbf{\Theta_{yy}}$ as finding the parameter values that satisfy the following optimality condition:

$$\frac{\partial L(\mathbf{\Theta_{xy}},\mathbf{\Theta_{yy}};\mathbf{X},\mathbf{Y})}{\partial \mathbf{\Theta_{yy}}} = \frac{1}{2}\mathbf{Y}^T\mathbf{Y} - \frac{1}{2}\sum_i E[\mathbf{y}^i\mathbf{y}^i T] = 0, \quad (9)$$

where the expectation is taken with respect to $p(\mathbf{y}^i|\mathbf{x}^i,\mathbf{\Theta_{xy}},\mathbf{\Theta_{yy}})$ and can be computed as

$$\begin{aligned} E[\mathbf{y}^i\mathbf{y}^i T] &= \mathrm{Cov}(\mathbf{y}^i) + E[\mathbf{y}^i]E[\mathbf{y}^i]^T \\ &= \mathbf{\Theta_{yy}}^{-1} + \mathbf{\Theta_{yy}}^{-1}\mathbf{\Theta_{xy}}^T\mathbf{x}^i\mathbf{x}^i T\mathbf{\Theta_{xy}}\mathbf{\Theta_{yy}}^{-1}. \end{aligned}$$

Then, from the above two equations, we obtain

$$\mathbf{Y}^T\mathbf{Y} = N\mathbf{\Theta_{yy}}^{-1} + \sum_i \mathbf{\Theta_{yy}}^{-1}\mathbf{\Theta_{xy}}^T\mathbf{x}^i\mathbf{x}^i T\mathbf{\Theta_{xy}}\mathbf{\Theta_{yy}}^{-1}. \quad (10)$$

The left-hand side of the above equation corresponds to the observed covariance of gene-expression traits, whereas the first and second terms on the right-hand side of the equation correspond to the decomposition of the overall covariance into the part induced by gene network and the other part induced by SNP perturbations, respectively. Eqs. (9) and (10) also follow directly from the standard result on the property of log-linear models, a type of undirected graphical models, that finding maximum likelihood estimates of parameters is equivalent to matching the expectation of features under the model with the empirical expectation of features (Theorem 20.1 in [29]). CGGMs are a member of log-linear models, and in Eq. (9), the expectation of features under the model and the empirical expectation of features correspond to $\sum_i E[\mathbf{y}^i\mathbf{y}^i T]$ and $\mathbf{Y}^T\mathbf{Y}$, respectively, where $y^i_{(m)}y^i_{(l)}$ are features. In sparse CGGMs, because of the bias introduced by the L_1 penalty for sparsity in parameters, the equality in Eq. (10) holds only approximately.

Relationship between CGGM and Standard Regression Method. The CGGM representation in Eq. (5) as obtained after inference in the probabilistic graphical model in Eq. (6) reveals its connection to the standard regression model in Eq. (2). The standard regression model is parameterized directly by $\mathbf{B}$ and $\mathbf{\Psi}$, whereas in CGGMs, the graphical model parameterized by $\mathbf{\Theta_{yy}}$ and $\mathbf{\Theta_{xy}}$ is learned first and then $\mathbf{B}$ and $\mathbf{\Psi}$ are obtained via inference. Maximum-likelihood estimation of parameters in the absence of sparsity constraint leads to the same estimate for $\mathbf{B}$ in both models. However, imposing a sparsity constraint on $\mathbf{\Theta_{xy}}$ and $\mathbf{\Theta_{yy}}$ for the conditional dependencies and then obtaining $\mathbf{B}$ via inference in sparse CGGM results in different estimates for $\mathbf{B}$, compared to sparsifying $\mathbf{B}$ directly as in the standard sparse regression approach. We argue that imposing a sparsity constraint on CGGM parameters rather than on the standard regression parameters produces more interpretable results that distinguish between sparse direct SNP perturbations and their propagation through the gene network for indirect SNP perturbations of the network. This is analogous to the case of Gaussian graphical models, where it is generally accepted as a more reasonable approach to impose a sparsity constraint on inverse covariance parameters for the graphical model than on covariance parameters that result from inference in the graphical model.

The CGGM representation in Eq. (5) after performing inference also reveals its connection to and advantages over MRCE. Although MRCE and CGGM are related in that $\mathbf{\Omega}$ in MRCE corresponds to $\mathbf{\Theta_{yy}}$ in CGGM, MRCE is based on the standard regression model and is parameterized by $\mathbf{B}$. One of the key advantages of using CGGM parameterization over MRCE parameterization is that our formulation leads to a convex optimization for estimation, whereas MRCE estimation problem is only bi-convex, not jointly convex. The important consequence of this difference is that MRCE optimization only reaches a locally-optimal solution with unstable convergence. In contrast, CGGM optimization is orders-of-magnitude more efficient than MRCE and finds the globally optimal solution, achieving higher accuracy for the recovered network and eQTLs with greater computational efficiency. Finally, although $\mathbf{\Omega}$ in MRCE and $\mathbf{\Theta_{yy}}$ in CGGM are equivalent as far as the model is concerned, their estimates are not identical because of the difference in the properties of optimization problems and the quality of solutions between the two approaches.

Results

In this section, we demonstrate the performance of our statistical framework on HapMap-simulated and yeast eQTL datasets [20,21] and compare the results with those from MRCE [22] that has been previously developed for genetical genomics analysis. Although our method primarily focuses on the recovery of gene network under SNP perturbations, it has the potential to enhance the power for detecting eQTLs by considering eQTL mapping in the context of network learning problem. In order to test this hypothesis, we also compare our method and MRCE with GFlasso [8] in terms of the accuracy for detecting eQTLs, as GFlasso has been developed specifically for eQTL mapping.

We used a Matlab implementation of Algorithm 1 in Text S2 for sparse CGGM, an R implementation of MRCE downloaded from the authors' website, and a Matlab implementation of the smoothing proximal gradient method [34] for GFlasso. We used the approximate MRCE algorithm that terminates the exact method after two iterations as suggested by the authors [22], because the exact method often did not converge and was too slow to be applicable to large-scale simulation experiments, taking two or three days with cross-validation for a single simulated dataset of 500 SNPs and 30 gene-expression traits.

Simulation Study

In order to simulate eQTL datasets, we used the SNP genotype data from HapMap phase III release 2 [20] as SNP data $\mathbf{X}$ and simulated gene-expression traits $\mathbf{Y}$, given $\mathbf{X}$ and known model parameters. We used the SNP data for chromosome 21 of the 343 individuals of African origin, including ASW, LWK, MKK, and YRI population groups. After removing SNPs with minor allele frequency <0.05 and highly correlated SNPs with squared correlation coefficient $r^2>0.6$, we obtained 4,901 SNPs. In each simulated dataset, we randomly selected a region of 500 SNPs and simulated the values of 30 gene-expression traits for each individual, based on the CGGM in Eq. (5). As almost all statistical methods for eQTL mapping assumes the standard linear regression model in Eq. (2), we performed experiments on gene-expression traits simulated from this model as well. In order to set the model parameters, we first set the sparsity pattern and then assigned values to the non-zero elements of the parameters as follows.

- *Simulation from CGGM with known* $\mathbf{\Theta}_{\mathbf{xy}}$ *and* $\mathbf{\Theta}_{\mathbf{yy}}$. In order to set the sparsity pattern of $\mathbf{\Theta}_{\mathbf{yy}}$ for gene network structure, we randomly partitioned the 30 expression traits into three modules of similar size and assumed relatively dense edge connections within each module and sparse edge connections between different modules. Specifically, we connected a pair of gene-expression traits with an edge with probability 0.5 within a module and with probability 0.02 between modules. Given this sparsity pattern in $\mathbf{\Theta}_{\mathbf{yy}}$, we assigned values to the non-zero elements of $\mathbf{\Theta}_{\mathbf{yy}}$ by drawing edge weights randomly from a uniform distribution $U[a,b]$ for pre-determined values of a and b and setting $\mathbf{\Theta}_{\mathbf{yy}}$ to the graph Laplacian of this weight matrix. A graph Laplacian is a symmetric matrix, where the off-diagonal elements are negative edge weights and diagonal elements are the sum of the weights of all the edges connecting to each node [35]. In general, a graph Laplacian is only positive-semidefinite, and to ensure $\mathbf{\Theta}_{\mathbf{yy}}$ is positive definite, we added small positive values to the diagonal elements.

- To assign the sparsity pattern of $\mathbf{\Theta}_{\mathbf{xy}}$ for direct SNP perturbations, we assumed that only a single gene-expression trait within each module is perturbed directly by a small number of SNPs. Specifically, we randomly selected one gene-expression trait in each of the three modules in $\mathbf{\Theta}_{\mathbf{yy}}$ and set each SNP as directly perturbing the selected gene-expression trait with probability 0.02. Then, we set the values of the non-zero elements of $\mathbf{\Theta}_{\mathbf{xy}}$ to random draws from a uniform distribution $U[a,b]$.
- *Simulation from the standard linear regression model with known* $\mathbf{B}$ *and* $\mathbf{\Psi}$. We used the same strategy as in $\mathbf{\Theta}_{\mathbf{yy}}$ to set $\mathbf{\Psi}^{-1}$. In order to set the sparsity pattern of $\mathbf{B}$, we assumed pleiotropic effects of eQTLs, where all gene-expression traits within each module are influenced by the same set of SNPs. For each gene module in $\mathbf{\Psi}^{-1}$, we let each SNP be an eQTL for the gene-expression traits in the module with probability 0.02. The association strengths for these eQTLs were randomly drawn from a uniform distribution $U[a,b]$.

While in our simulation studies we primarily focused on the relatively small datasets of 500 SNPs and 30 gene-expression traits as described above, in order to demonstrate the performance and scalability of our method, we also applied our method to larger-scale simulated datasets of 1,000 SNPs and 500 gene-expression traits. Because MRCE, the main competing method to our approach, required substantially more computation time than our approach even on the smaller datasets, we were unable to compare the performance of MRCE on these larger simulated datasets. Instead, we compared our method with other computationally efficient methods, including GFlasso and a base-line approach of applying graphical lasso [16] and lasso [25] sequentially to learn gene networks and eQTLs.

We use precision-recall curves and prediction errors as quantitative measures of the performance of different statistical methods. Precision-recall curves summarize how accurately each method recovers the true eQTLs and gene network structure by plotting precisions and recalls on y- and x-axes. In order to compute precisions and recalls, for each simulated dataset, we ranked all the elements of the estimated parameter matrices in a descending order according to their absolute values and compared this ranked list with the set of non-zero elements in the true parameters. On the other hand, prediction errors evaluate the performance of different methods on how accurately each method can predict gene-expression levels, given SNPs and estimated model parameters. Once the parameters are estimated using training data, prediction errors are obtained as $\frac{1}{N_{\mathrm{ts}}K}\sum_i (\mathbf{y}^i-\hat{\mathbf{y}}^i)^T(\mathbf{y}^i-\hat{\mathbf{y}}^i)$, where $\hat{\mathbf{y}}^i$ is the prediction of gene expressions given by the model for the ith individual in an independent test dataset and N_{ts} is the number of samples in the test set.

Given the 343 samples in the full dataset, we used 300 samples as a training dataset and the remaining 43 samples as a test dataset. In order to determine the optimal regularization parameters in sparse CGGM, MRCE, and GFlasso during the training phase, we created a grid for different choices of regularization parameters, performed a five-fold cross-validation for each point on the grid, and selected the values that give the smallest cross-validation error as the optimal regularization parameters.

Illustration of Sparse CGGM Behavior

In order to illustrate the behavior of a sparse CGGM, we present the results from applying our method to a single dataset simulated from a CGGM parameterization and compare them with what we obtained from GFlasso and MRCE (Figure 2). The non-zero elements of the true model parameters were drawn from $U[0.8,1.1]$ for $\mathbf{\Theta}_{\mathbf{xy}}$ and $U[0.2,0.5]$ for $\mathbf{\Theta}_{\mathbf{yy}}$. Since GFlasso requires the gene network to be known, we used the correlation coefficient matrix of gene-expression trait data thresholded at 0.6 as a gene network in GFlasso estimation. The true parameters for $\mathbf{\Theta}_{\mathbf{yy}}$ and $\mathbf{\Theta}_{\mathbf{xy}}$ along with $\mathbf{B}=-\mathbf{\Theta}_{\mathbf{xy}}\mathbf{\Theta}_{\mathbf{yy}}^{-1}$ are shown in the left, middle, and right columns, respectively, in Figure 2A. The estimated parameters for sparse CGGMs, MRCE, and GFlasso are shown in Figures 2B–D, respectively. In the plots for $\mathbf{\Theta}_{\mathbf{xy}}$ and $\mathbf{B}$, the rows and columns correspond to gene-expression traits and SNPs, respectively, and the results are shown only for the first 150 SNPs. In each panel, the white pixels correspond to the zero elements of the parameters and the darker pixels to non-zero elements. We note that while sparse CGGM provides the estimates of both $\mathbf{\Theta}_{\mathbf{xy}}$ and $\mathbf{B}$, MRCE and GFlasso provide a single estimate of eQTL effect sizes in $\mathbf{B}$ and do not distinguish between direct and indirect effects of eQTLs on gene-expression traits.

As shown in Figure 2B, our method successfully recovers the three gene modules in the true gene network as the block-diagonal structure in $\mathbf{\Theta}_{\mathbf{yy}}$ along with the sparse direct perturbation of this network by eQTLs in $\mathbf{\Theta}_{\mathbf{xy}}$. When we perform inference in the estimated sparse CGGM by computing $\mathbf{B}=-\mathbf{\Theta}_{\mathbf{xy}}\mathbf{\Theta}_{\mathbf{yy}}^{-1}$ to learn indirect perturbation of the network by eQTLs, the direct perturbations of eQTLs in $\mathbf{\Theta}_{\mathbf{xy}}$ propagate primarily within each gene module in $\mathbf{\Theta}_{\mathbf{yy}}$, leading to vertical stripes in $\mathbf{B}$. Although MRCE learns a gene network from data, unlike sparse CGGM, it does not have any mechanism to leverage this gene network to learn pleiotropic or indirect effects of eQTLs on gene modules and the estimated $\mathbf{B}$ in Figure 2C shows isolated eQTLs for individual gene-expression traits rather than vertical stripes. As can be seen in Figure 2D, the GFlasso estimate of $\mathbf{B}$ shows vertical stripes for eQTLs common within each gene module. However, it is immediately clear that GFlasso results have significantly more false positives for eQTLs than sparse CGGM and MRCE. This demonstrates that genetical genomics approach has the potential to improve the accuracy for detecting eQTLs than the conventional approach that focuses solely on eQTL mapping.

Simulation from Sparse CGGMs

We performed a quantitative comparison of the performance of sparse CGGM, MRCE, and GFlasso, by obtaining precision-recall curves and prediction errors averaged over 50 simulated datasets. Since MRCE and GFlasso are based on the standard linear

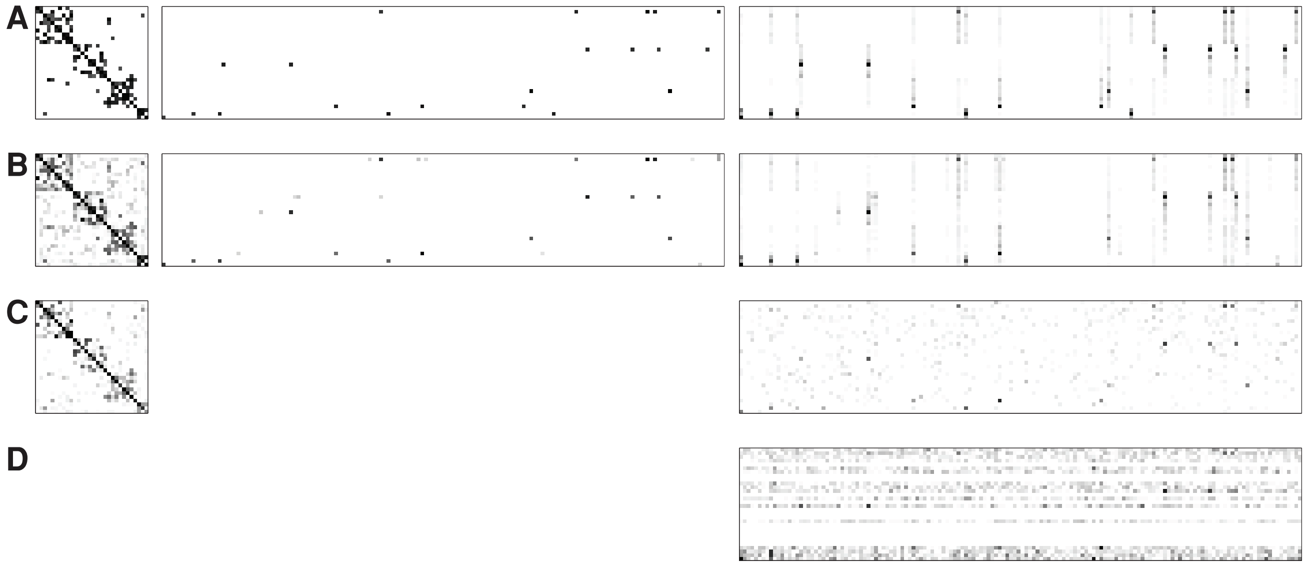

Figure 2. Comparison of the behavior of sparse CGGM, MRCE, and GFlasso using a single simulated dataset. A known sparse CGGM was used to generate the simulated dataset. The left, middle, and right columns show the absolute values of $\boldsymbol{\Theta}_{\mathbf{yy}}$ for gene-network edge weights, $\boldsymbol{\Theta}_{\mathbf{xy}}$ for the strengths of direct SNP perturbations, and $\mathbf{B} = -\boldsymbol{\Theta}_{\mathbf{yy}}^{-1}\boldsymbol{\Theta}_{\mathbf{xy}}^{T}$ for strengths of indirect SNP perturbations, respectively. In the middle and right columns, $\boldsymbol{\Theta}_{\mathbf{xy}}$ and $\mathbf{B}$ are shown with gene-expression traits in rows and SNPs in columns. White pixels represent zero elements and darker pixels represent non-zero elements of the parameter matrix. The true model parameters are shown in Panel (A), and the estimated parameters are shown for (B) sparse CGGM, (C) MRCE, and (D) GFlasso. MRCE and GFlasso use the standard regression model for eQTL mapping, and thus provide a single summary of SNP effects on gene expressions in $\mathbf{B}$. GFlasso focuses only on the task of eQTL mapping and thus, does not provide an estimate of gene network.
doi:10.1371/journal.pcbi.1003420.g002

regression model and sparse CGGM is based on a graphical model, we evaluate the different methods on datasets simulated from both models.

Recovery of Gene Networks. We first evaluate the two statistical methods, sparse CGGM and MRCE, on the accuracy of gene network structure recovery. The precision-recall curves for the estimated $\boldsymbol{\Theta}_{\mathbf{yy}}$ and $\boldsymbol{\Psi}^{-1}$ averaged over 50 simulated datasets are shown in Figure 3. In each panel of Figure 3, the true model parameters were drawn from one of the nine combinations of the three uniform distributions, namely $U[0.2,0.5]$, $U[0.5,0.8]$, and $U[0.8,1.1]$ for $\boldsymbol{\Theta}_{\mathbf{xy}}$ (rows in Figure 3) and $\boldsymbol{\Theta}_{\mathbf{yy}}$ (columns in Figure 3). The results in Figure 3 show that sparse CGGMs can recover the gene network structure with significantly higher accuracy for nearly all of the parameter settings. In addition, the performance of sparse CGGMs is relatively stable across all parameter settings, whereas the performance of MRCE varies widely for different parameter settings.

Recovery of eQTLs. In the process of identifying the gene network structure under SNP perturbations, sparse CGGM and MRCE also address eQTL mapping, identifying the SNPs with or without perturbation effects on gene network. Using the same simulated datasets and estimated models from Figure 3, in Figure 4, we evaluate the two methods on how accurately they can recover the true eQTLs, and compare the precision-recall curves with those from GFlasso that focuses only on eQTL mapping. For sparse CGGM, we present two precision-recall curves, one for direct eQTL effects in $\boldsymbol{\Theta}_{\mathbf{xy}}$ and another for indirect eQTL effects obtained from $\mathbf{B} = -\boldsymbol{\Theta}_{\mathbf{xy}}\boldsymbol{\Theta}_{\mathbf{yy}}^{-1}$, by comparing the estimated values with the true $\boldsymbol{\Theta}_{\mathbf{xy}}$ and $\mathbf{B}$. For MRCE and GFlasso that do not distinguish between the direct and indirect effects of eQTLs, we obtained a single precision-recall curve by comparing the estimated $\mathbf{B}$ with the true $\mathbf{B} = -\boldsymbol{\Theta}_{\mathbf{xy}}\boldsymbol{\Theta}_{\mathbf{yy}}^{-1}$.

Overall, in Figure 4, sparse CGGM consistently outperforms MRCE and GFlasso on estimating eQTLs under all parameter settings. In addition, the two statistical methods for genetical genomics approach achieve significantly higher accuracy for detecting eQTLs than GFlasso that focuses on eQTL mapping, showing the advantage of discovering eQTLs by systematically decoding a gene network and its perturbation effects by SNPs. We also observe that for a given parameter setting for $\boldsymbol{\Theta}_{\mathbf{yy}}$ in each column of Figure 4, as the range of values for $\boldsymbol{\Theta}_{\mathbf{xy}}$ increases from $U[0.2,0.5]$ to $U[0.8,1.1]$, the performance of all methods on detecting eQTLs tend to improve.

Prediction Errors. In order to evaluate the different methods on how accurately the estimated model can predict gene-expression values given SNPs, we computed the prediction errors on the test data using the same set of estimated models in Figures 3 and 4. The results are shown as box plots in Figure 5. We observe from Figure 5 that sparse CGGMs consistently outperform MRCE and GFlasso with the smallest mean and variance of the prediction errors under all of the nine parameter settings.

Simulation from Standard Linear Regression Models

Since MRCE and GFlasso use the standard linear regression model, we also compared the performance of the different methods, using datasets simulated from the model in Eq. (2) with known parameters for $\mathbf{B}$ and $\boldsymbol{\Psi}$ (Figure 6). We present the precision-recall curves for $\boldsymbol{\Psi}$ and $\mathbf{B}$ averaged over 50 simulated datasets in Figures 6A and B, respectively, and show the prediction errors in Figure 6C. In our simulation, we set the true parameter values to random draws from $U[0.5,0.8]$ for $\boldsymbol{\Psi}$ and $U[0.5,0.8]$ for $\mathbf{B}$. As can be seen in Figure 6, even if the datasets were simulated from the standard linear regression model as used in MRCE and GFlasso, our method still outperforms MRCE and GFlasso.

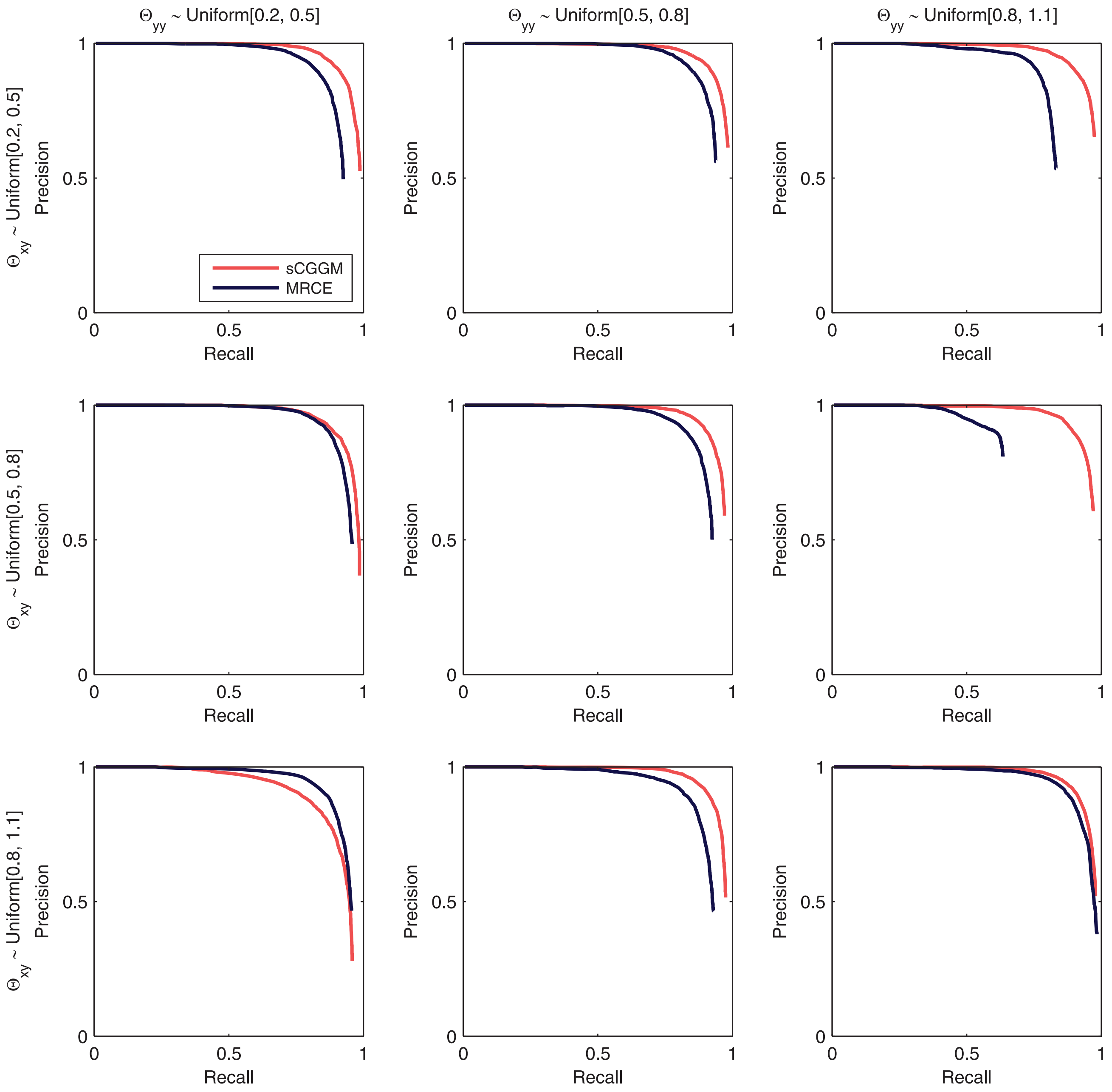

Figure 3. Precision-recall curves for estimated gene network structures using datasets simulated from sparse CGGMs. Each panel shows the results from datasets simulated under different parameter settings for $\boldsymbol{\Theta}_{\mathbf{xy}}$ (rows) and $\boldsymbol{\Theta}_{\mathbf{yy}}$ (columns). Each precision-recall curve was obtained as an average over results from 50 simulated datasets. Simulated datasets with 30 gene-expression traits and 500 SNPs were used.
doi:10.1371/journal.pcbi.1003420.g003

Simulation Using Large-Scale Datasets

The simulation results so far demonstrated that our method has greater power for identifying gene networks and eQTLs than other methods. However, in these experiments, we were constrained to use relatively small datasets of only 30 gene-expression traits with 500 SNPs, because MRCE could not handle larger datasets effectively in a systematic simulation study. In this section, we demonstrate the effectiveness and scalability of our method, using substantially larger simulated datasets of 500 gene-expression traits and 1,000 SNPs.

Given a region of 1,000 SNPs from chromosome 21 of the African individuals in HapMap phase III SNP data as described above, we simulated the values for 500 gene-expression traits, assuming sparse CGGMs with the true parameters determined as follows. We assumed that the true gene networks are scale-free networks, and set the network using the following strategy. First, we determined the number of neighbors of each node by making a random draw from a power-law distribution $p(k) \propto k^{-1.8}$. Then, we applied the algorithm for generating a scale-free network [36] that repeatedly connects two nodes until we achieve the desired node degrees initially determined according to the power-law distribution. Given this network structure, we set the edge weights to random draws from a uniform distribution $U[0.5,0.8]$, and set $\boldsymbol{\Theta}_{\mathbf{yy}}$ to the graph Laplacian of the edge-weight matrix with small

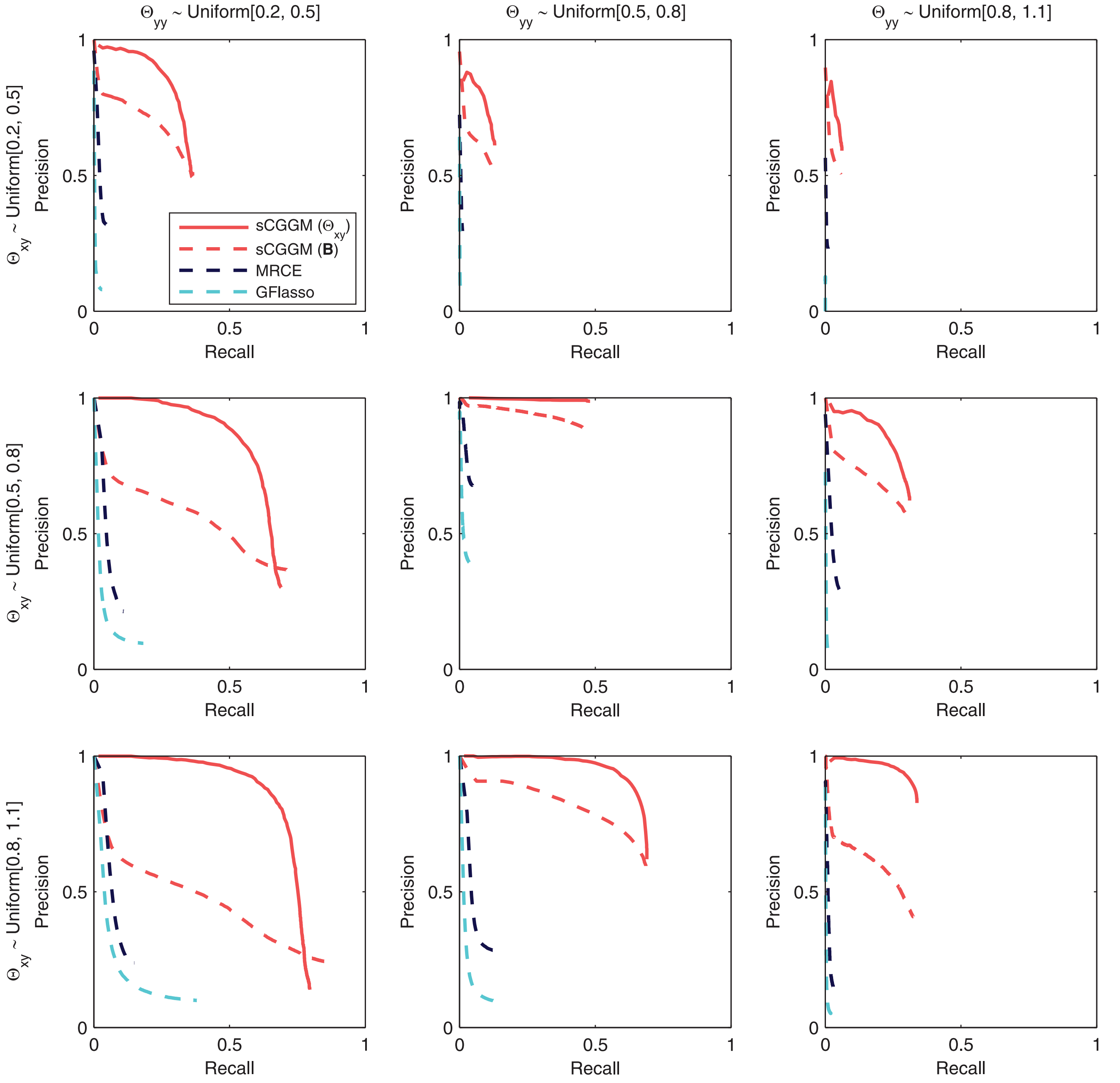

Figure 4. Precision-recall curves for estimated eQTLs using datasets simulated from sparse CGGMs. Precision-recall curves for the recovery of eQTLs are shown, using the same simulated datasets and estimated models in Figure 3. Each panel shows results from datasets simulated under different parameter settings for $\boldsymbol{\Theta}_{\mathbf{xy}}$ (rows) and $\boldsymbol{\Theta}_{\mathbf{yy}}$ (columns). For sparse CGGMs, each panel shows two precision-recall curves, one for eQTLs with direct perturbation effects $\boldsymbol{\Theta}_{\mathbf{xy}}$ and another for indirect perturbation effects $\mathbf{B}$, whereas for MRCE and GFlasso, the results are shown only for the association strengths $\mathbf{B}$.
doi:10.1371/journal.pcbi.1003420.g004

positive values added to the diagonal elements. We set the true eQTLs in $\boldsymbol{\Theta}_{\mathbf{xy}}$ by choosing each SNP as an eQTL for each gene-expression trait with probability 0.02 and selecting one additional SNP as an eQTL for hub nodes with more than 20 neighbors in the network. For each eQTL in $\boldsymbol{\Theta}_{\mathbf{xy}}$, we set the eQTL effect sizes to random draws from a uniform distribution $U[0.5,0.8]$ with the signs of the values determined randomly.

In Figure 7, we compare the performance of the different methods averaged over 30 datasets simulated according to the above strategy. The results are shown for the precision-recall curves for the accuracy of detecting gene-network structures (Figure 7A) and eQTLs (Figure 7B) as well as prediction errors (Figure 7C). As MRCE could not run on a single dataset of the given size within a few days, instead of using MRCE in our experiment, we compared our method with GFlasso and also with a two-stage method of applying graphical lasso and lasso to learn gene networks and eQTLs separately. We observe from Figure 7 that sparse CGGMs outperform all the other methods on these large-scale datasets.

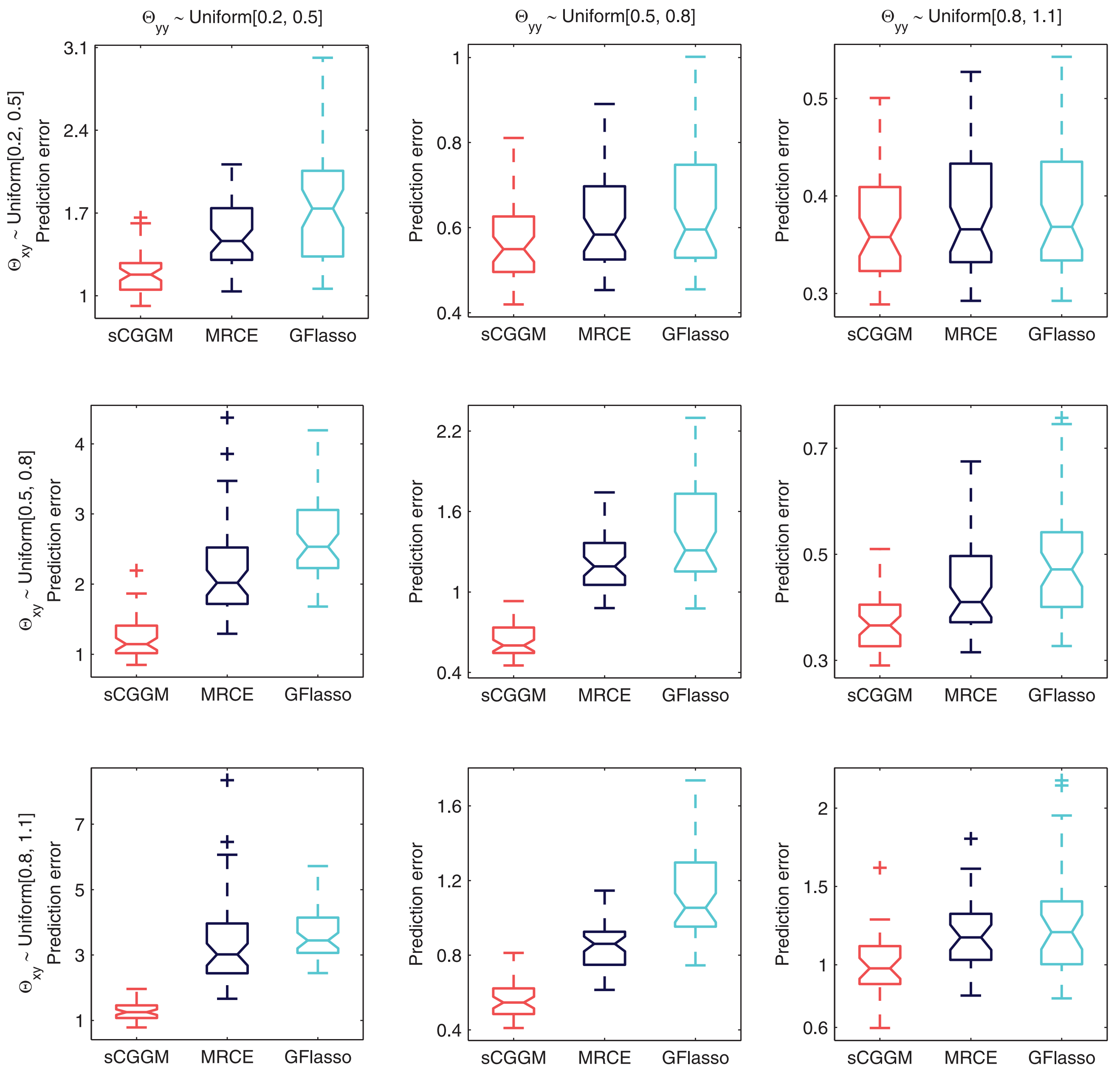

Figure 5. Prediction errors in simulation studies. Prediction errors on an independent test data given the estimated models in Figures 3 and 4 are shown as boxplots for different parameter settings for $\mathbf{\Theta_{xy}}$ (rows) and $\mathbf{\Theta_{yy}}$ (columns).
doi:10.1371/journal.pcbi.1003420.g005

Computation Time and Scalability

In order to examine the scalability of sparse CGGM, MRCE, and GFlasso, we compared the computation time for a single run of the different methods on varying sizes of datasets in Figure 8. Figure 8A shows the computation time for varying the number of gene-expression traits with the number of SNPs fixed at $J=300$, whereas Figure 8B shows the results from varying the number of SNPs with the number of gene-expression traits fixed at $K=100$. Even though we used the approximate method for MRCE to reduce the computational cost of the exact method, our sparse CGGM optimization is more efficient by orders of magnitude than MRCE for problems of all sizes. Although GFlasso is more efficient than both sparse CGGM and MRCE, it is significantly more limited in that it focuses only on the problem of eQTL mapping.

Analysis of Yeast eQTL Dataset

We applied our method to an eQTL dataset collected for two yeast parent strains, BY4716 (BY) and RM11-1a (RM), and their 112 segregants [21]. We obtained SNP genotypes for 1,260 loci after removing the redundant SNPs with the same genotypes in neighboring regions of the genome and obtained expression measurements for 3,684 genes after removing the genes whose expression measurements were missing for more than 5% of the 114 samples. In order to select the optimal regularization parameters λ_1 and λ_2, we performed a cross-validation with three random splits of data into 100 samples for estimating model parameters and 14 samples for computing cross-validation errors. Then, a final estimate of parameters was obtained by training a model on the entire dataset using the optimal regularization parameters.

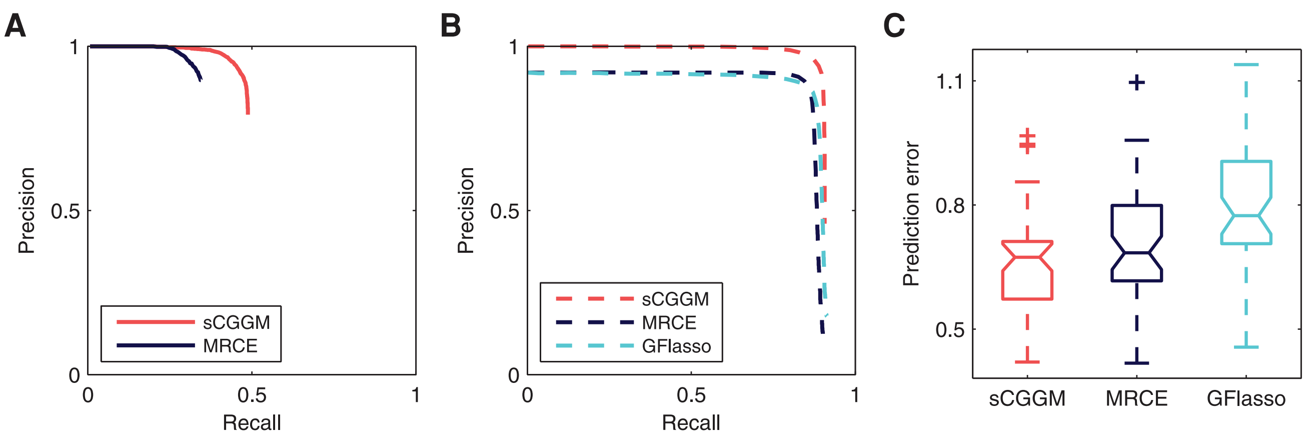

Figure 6. Results from datasets simulated from the standard linear regression model. (A) Precision-recall curves for the recovery of gene network structure in Θ_{yy} (or Ψ^{-1}). (B) Precision-recall curves for the recovery of eQTLs in $\mathbf{B}$. (C) Prediction errors. The results were obtained as an average over 50 simulated datasets. Simulated datasets with 30 gene-expression traits and 500 SNPs were used.
doi:10.1371/journal.pcbi.1003420.g006

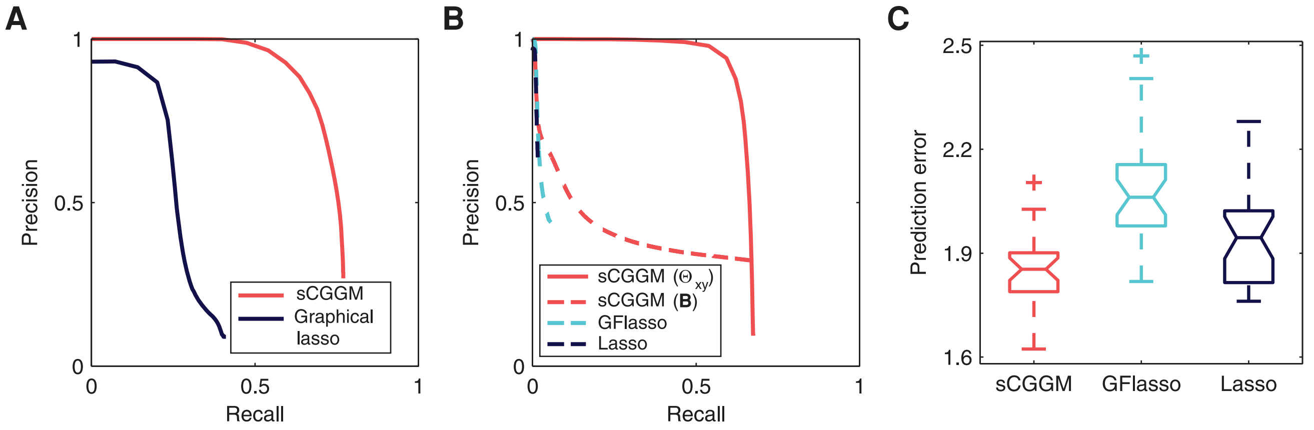

Figure 7. Results from large-scale datasets simulated with sparse CGGMs. (A) Precision-recall curves for the recovery of gene network structure in Θ_{yy} (or Ψ^{-1}). (B) Precision-recall curves for the recovery of eQTLs in $\mathbf{B}$. (C) Prediction errors. The results were obtained as an average over 30 simulated datasets. Simulated datasets with 500 gene-expression traits and 1,000 SNPs were used.
doi:10.1371/journal.pcbi.1003420.g007

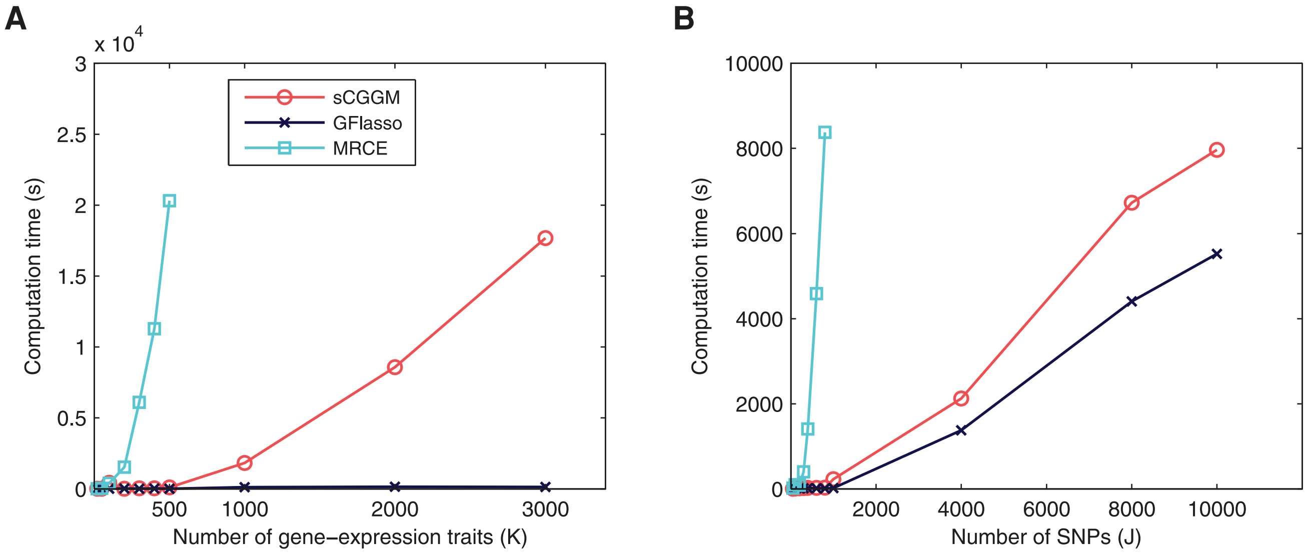

Figure 8. Computation time and scalability. The computation time for a single run of sparse CGGM, MRCE, and GFlasso is shown for (A) varying the number of gene-expression traits K with the number of SNPs fixed at $J=300$ and (B) varying the number of SNPs J with the number of gene-expression traits fixed at $K=100$. The results for MRCE were obtained using the approximate algorithm.
doi:10.1371/journal.pcbi.1003420.g008

Table 1. Yeast hub genes and hub-gene regulated modules estimated by sparse CGGM.

Hub gene	Hub module size	GO category	$p-$value*	Category size (Overlap)
CTT1	739	Cytoplasmic translation	3.08e-62	171 (110)
ARG1	564	Ribosome	2.24e-51	258 (111)
DSE2	340	Cytoplasmic translation	1.16e-22	171 (48)
PHM7	237	Carboxylic acid biosynthetic process	4.41e-15	392 (50)
GPH1	184	Ribonucleoprotein complex biogenesis	3.61e-26	192 (42)
MSC1	182	Oxidation-reduction process	5.11e-11	408 (38)
URA10	178	Cytoplasmic translation	1.53e-98	171 (88)
RTN2	150	Oxidation-reduction process	1.13e-11	408 (35)
ICY2	145	Cellular amino acid biosynthetic process	1.83e-27	153 (36)
SOL4	135	Oxidation-reduction process	1.28e-9	408 (30)
SPI1	119	Trehalose metabolic process	2.21e-10	11 (7)
		Glycoside metabolic process	2.19e-9	14 (7)
ASP3-3	118	Glutamine family amino acid catabolic process	1.06e-7	14 (6)
LEU2	104	Cellular amine metabolic process	2.37e-9	299 (22)
TKL2	105	Oxidation-reduction process	1.50e-7	408 (23)
HIS4	97	Cellular amino acid biosynthetic process	9.00e-56	153 (55)
YHR033W	93	Mitochondrial membrane part	1.84e-30	143 (32)
		Hydrogen ion transmembrane transporter activity	3.86e-20	57 (18)
		Respiratory electron transport chain	1.82e-18	31 (14)
		Cellular respiration	2.48e-18	70 (18)
SNZ1	91	Cellular amino acid biosynthetic process	8.43e-30	153 (32)
GAD1	91	Carbohydrate catabolic process	2.43e-8	84 (11)
		Trehalose metabolic process	2.42e-7	11 (5)
ARG3	90	Ribonucleoprotein complex biogenesis	2.41e-25	192 (31)
HSP26	87	Extra cellular region	4.73e-9	95 (12)
TMT1	75	Cellular amino acid biosynthetic process	1.09e-22	153 (25)
TFS1	67	Carbohydrate catabolic process	1.37e-8	84 (10)
		Trehalose metabolic process	5.13e-8	11 (5)
LYS1	62	Cellular amino acid metabolic process	1.79e-27	283 (32)
PGM2	57	Carbohydrate metabolic process	2.04e-11	283 (18)
		Energy reserve metabolic process	9.94e-11	29 (8)
		Glycogen biosynthetic process	1.76e-10	12 (6)
URA3	56	Superoxide dismutase activity	1.34e-5	6 (3)
		De novo pyrimidine base biosynthetic process	2.39e-5	7 (3)
		Response to toxin	2.39e-5	7 (3)

*The $p-$values were obtained from Fisher's exact test. Only the top GO categories with the most significant $p-$values are shown for each module.
doi:10.1371/journal.pcbi.1003420.t001

Below, we examine the yeast gene network and eQTLs with direct and indirect perturbations estimated by our method. In addition, we provide an in-depth analysis of a subnetwork with a strong evidence of being involved in DNA replication stress response based on the literature. We also provide a quantitative comparison of sparse CGGMs and other methods in terms of prediction errors.

Scale-Free Gene Network for Yeast

Since many previous works showed that gene networks tend to have a scale-free topology with few hub genes having many neighbors, we examined the gene network parameters $\mathbf{\Theta_{yy}}$ in the estimated sparse CGGM for a scale-free property [37]. Given the network edge weights in $\mathbf{\Theta_{yy}}$, we defined the degree of each node as the sum of the absolute values of all incoming edge weights for the node. Then, the best ordinary least square fit of linear model for the empirical cumulative degree distribution was $r^2=0.98$, showing that the estimated network has a strongly scale-free topology. Overall, in our estimate of network, 14 genes were connected to more than 100 other genes, 156 genes had more than 10 neighbors, and 1,593 genes had at least one neighbor.

We hypothesized that each hub gene and its immediate neighbors form a hub-gene module and are involved in a common biological process. In order to test this hypothesis, we performed a gene ontology (GO) enrichment analysis for the 25 largest hub-gene modules, using Fisher's exact tests (Table 1). We found that

Table 2. Yeast eQTL hotspots and hotspot-regulated modules in Θ_{xy} estimated by sparse CGGM.

Genome location	Hotspot module size	Slim GO category	$p-$value*	Category size (Overlap)	Zhu et al. [38]
II:368,991	146	Nucleolus	1.63e-21	124 (43)	
		RNA modification	1.69e-5	41 (11)	
II:548,401	359	Nucleolus	5.65e-24	124 (71)	√
		rRNA processing	4.71e-19	127 (63)	
		Ribosomal biogenesis	4.48e-10	58 (30)	
II:658,746	231	Nucleolus	2.75e-33	124 (71)	
		rRNA processing	3.14e-25	127 (60)	
III:105,042	77	Cellular amino acid metabolic process	5.95e-8	148 (18)	
III:79,091	75	Oxidoreductase activity	1.84e-6	152 (16)	√
IV:871,416	64	Response to chemical stimulus	6.25e-4	125 (10)	
IV:929,769	105	Mitochondrial translation	1.75e-10	23 (10)	
V:113,507	103	Plasma membrane	3.38e-7	124 (18)	√
		Transmembrane transporter activity	2.63e-5	109 (14)	
V:350,744	174	Structural molecule activity	4.00e-20	144 (48)	
V:395,442	79	Cellular respiration	2.20e-7	50 (11)	
		Nucleic acid binding transcription factor activity	1.92e-5	39 (8)	
VII:52,613	109	Endoplasmic reticulum	3.43e-6	99 (15)	
VIII:89,953	63	Chromatin organization	1.29e-7	27 (8)	√
VIII:111,686	67	Conjugation	8.00e-11	27 (11)	√
		Site of polarized growth	3.47e-7	40 (9)	
		Regulation of cell cycle	1.13e-5	33 (7)	
		Cellular bud	2.99e-5	39 (7)	
VIII:138,639	64	Mitochondrial translation	1.32e-7	23 (7)	
		Mitochondrion organization	1.17e-6	68 (9)	
XII:957,108	61	Cellular respiration	1.29e-13	50 (16)	
XII:660,992	196	Oxidoreductase activity	3.95e-13	152 (34)	√
		Lipid metabolic process	2.71e-7	76 (17)	
		Response to chemical stimulus	1.09e-5	125 (19)	
XIII:46,077	86	Cellular amino acid metabolic process	8.30e-6	148 (15)	
		Carbohydrate metabolic process	1.63e-5	85 (11)	
XIV:486,861	410	Mitochondrion organization	2.85e-7	68 (21)	√
		Mitochondrial envelope	1.03e-6	118 (27)	
		Generation of precursor metabolites	2.95e-6	80 (21)	
XV:172,654	249	Oxidoreductase activity	1.48e-10	152 (38)	√
		Mitochondrial envelope	6.60e-10	118 (32)	
XV:563,943	107	Generation of precursor metabolites	4.75e-27	80 (37)	√
		Cellular respiration	4.32e-26	50 (31)	
		Ion transport	1.29e-9	71 (17)	
		ATPase activity	3.45e-5	68 (11)	

*The p-values were obtained from Fisher's exact test. Only the top GO categories with the most significant $p-$values are shown for each module.
doi:10.1371/journal.pcbi.1003420.t002

each hub-gene module was significantly enriched with genes in a common GO category, showing that the genes in each hub-gene module are likely to participate in a common pathway.

SNP Perturbations of Yeast Gene Network

Next, we examined the eQTLs identified by our method as perturbing the above gene network. The eQTLs with direct perturbations of the gene network as captured in Θ_{xy} tended to concentrate on a small number of genetic loci, forming eQTL hotspots. Although the sparsity pattern of Θ_{xy} showed that 1,248 out of 1,260 SNP loci regulate directly at least one gene-expression trait, the top 10 SNPs that affect the largest number of gene-expression traits accounted for 15.8% of all SNP/gene-expression-trait pairs with direct influence of SNPs on gene-expression traits, and the top 20 SNPs accounted for 28.5%. We defined these top 20 SNPs as eQTL hotspots, and the genes directly regulated by each eQTL

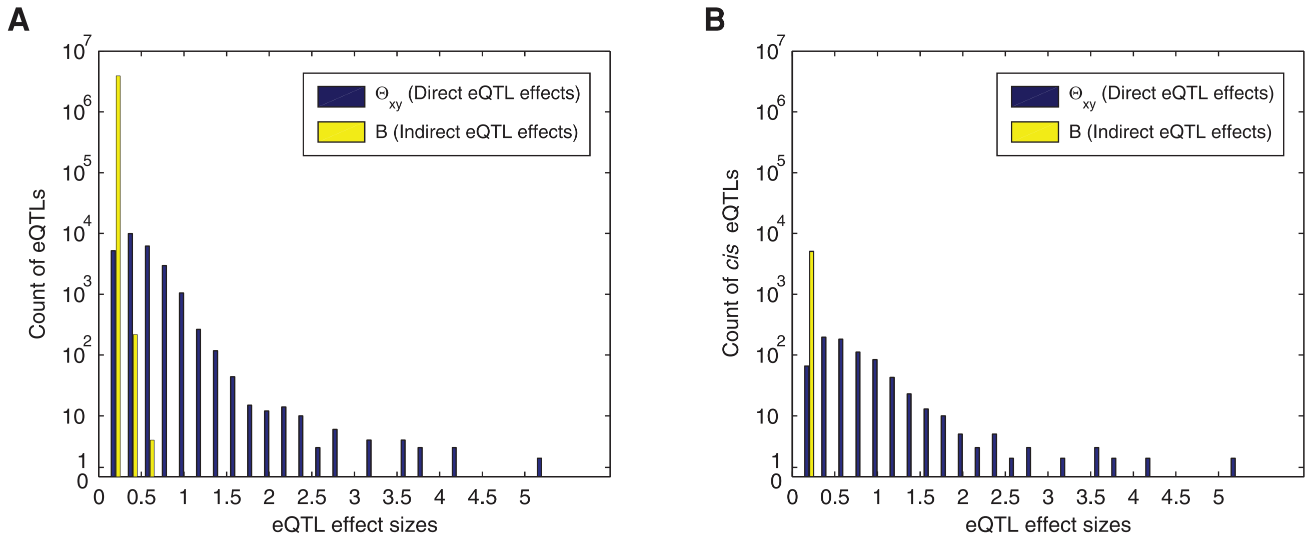

Figure 9. Comparison of SNP perturbation effect sizes on yeast gene network in the estimated sparse CGGM. Histograms of the effect sizes of direct and indirect SNP perturbations in yeast are shown for (A) all eQTLs and (B) *cis* eQTLs identified by sparse CGGM.
doi:10.1371/journal.pcbi.1003420.g009

hotspot as a hotspot-regulated gene module (Table 2). In order to avoid redundancy, in the case of multiple eQTL hotspots within a 20 kb region with largely overlapping hotspot-regulated gene modules, we examined only one of those hotspots with the largest hotspot-regulated gene module. Out of the 13 eQTL hotspots that have been previously reported in analysis of the same dataset in [38], 9 hotspots overlapped with the results from our method.

In order to investigate whether the genes in each hotspot-regulated gene module are involved in a common biological function, we performed GO enrichment analysis (Table 2). We performed Fisher's exact tests, using GO slim categories downloaded from http://www.geneontology.org/GO.slims.shtml, after removing the GO categories with more than 500 genes. Our results show that all of the hotspot-regulated gene modules are significantly enriched for some GO categories, providing evidence that each eQTL hotspot regulates a functionally coherent set of genes.

Since indirect SNP perturbations result from direct SNP perturbations propagating through the network, eQTLs with direct perturbations are likely to have stronger effect sizes than indirect perturbations. In Figure 9A, we compared the overall distribution of effect sizes of direct and indirect SNP perturbations as captured in $\mathbf{\Theta_{xy}}$ and $\mathbf{B}$ of the sparse CGGM by plotting histograms of the absolute values of non-zero elements in $\mathbf{\Theta_{xy}}$ and $\mathbf{B}$. For indirect SNP perturbations, only those SNP/gene-expression-trait pairs that were estimated to be zero in $\mathbf{\Theta_{xy}}$ but non-zero valued in $\mathbf{B}$ were included. As can be seen in Figure 9A, the direct perturbations are generally stronger than indirect perturbations, confirming our hypothesis.

Then, we examined whether the direct SNP perturbations estimated by our method are more likely to be *cis* eQTLs than the indirect perturbations. We declared the direct and indirect SNP perturbations in $\mathbf{\Theta_{xy}}$ and $\mathbf{B}$ as *cis* eQTLs, if for a given pair of SNP/gene-expression-trait, the gene sequence overlaps with the linkage region represented by the given SNP. The histogram in Figure 9B shows the distribution of the effect sizes of the estimated direct and indirect SNP perturbations for *cis* eQTLs. As can be seen in Figure 9B, direct SNP perturbations are significantly more frequent in *cis* eQTLs than indirect SNP perturbations, and explain nearly all of the *cis* eQTLs with strong effect sizes.

When we examined the *cis* eQTLs with direct perturbations in our estimated model, we found that our approach was able to identify some of the well-known direct genetic perturbations in the literature. The genotypes for *LEU2*, *URA3*, *HO*, and *LYS2* are known to differ in the parent strains, BY and RM, where these genetic differences have a large impact on the expressions of the corresponding genes as well as other genes [21]. While *LYS2* was not included in our analysis, the *LEU2*, *URA3*, and *HO* expressions were found to have *cis* eQTLs with direct perturbations in our analysis, and at the same time, in our estimate of gene network, *LEU2* and *URA3* appeared as hub genes with more than 50 neighbors. In particular, the *cis* eQTLs with direct perturbations of *LEU2* and *URA3* were found to have the strongest effects among all *cis* eQTLs shown in Figure 9B, whereas *HO* had a *cis* eQTL with moderately strong direct perturbation. These results provide evidence that our method can recover the true direct SNP perturbations of a gene network, by decoupling direct SNP perturbations of gene expressions from their secondary/indirect effects on other gene expressions.

DNA Replication Stress Response Subnetwork and its Perturbation by eQTLs in Yeast

We performed an in-depth analysis of the subnetwork around the *TFS1* gene and its perturbation by eQTLs. This subnetwork is shown in Figure 10A, where the edge thicknesses correspond to the absolute values of edge weights in $\mathbf{\Theta_{yy}}$, representing the strength of dependency between two gene-expression traits. To avoid clutter, we only show the edges with the absolute values of edge weights >0.05.

This subnetwork in Figure 10A contains many genes involved in DNA replication stress response and other types of stimulus response. In particular, *TFS1* has been identified as a high-copy suppressor of guanine nucleotide-exchange factor *CDC25*, which activates the Ras/cyclic AMP pathway regulating growth and metabolism in response to nutrients [39]. Many of the genes in this subnetwork, including *TFS1*, its immediate neighbors (*PGM2*, *SOL4*, *RTN2*, *GDB1*, *RME1*, *PRB1*, *SDS24*, *IGD1*), and 22 other genes, have been previously observed with changed abundance or localization under DNA damage [40]. In addition, the *DDR2* gene in the subnetwork that codes for the DNA damage

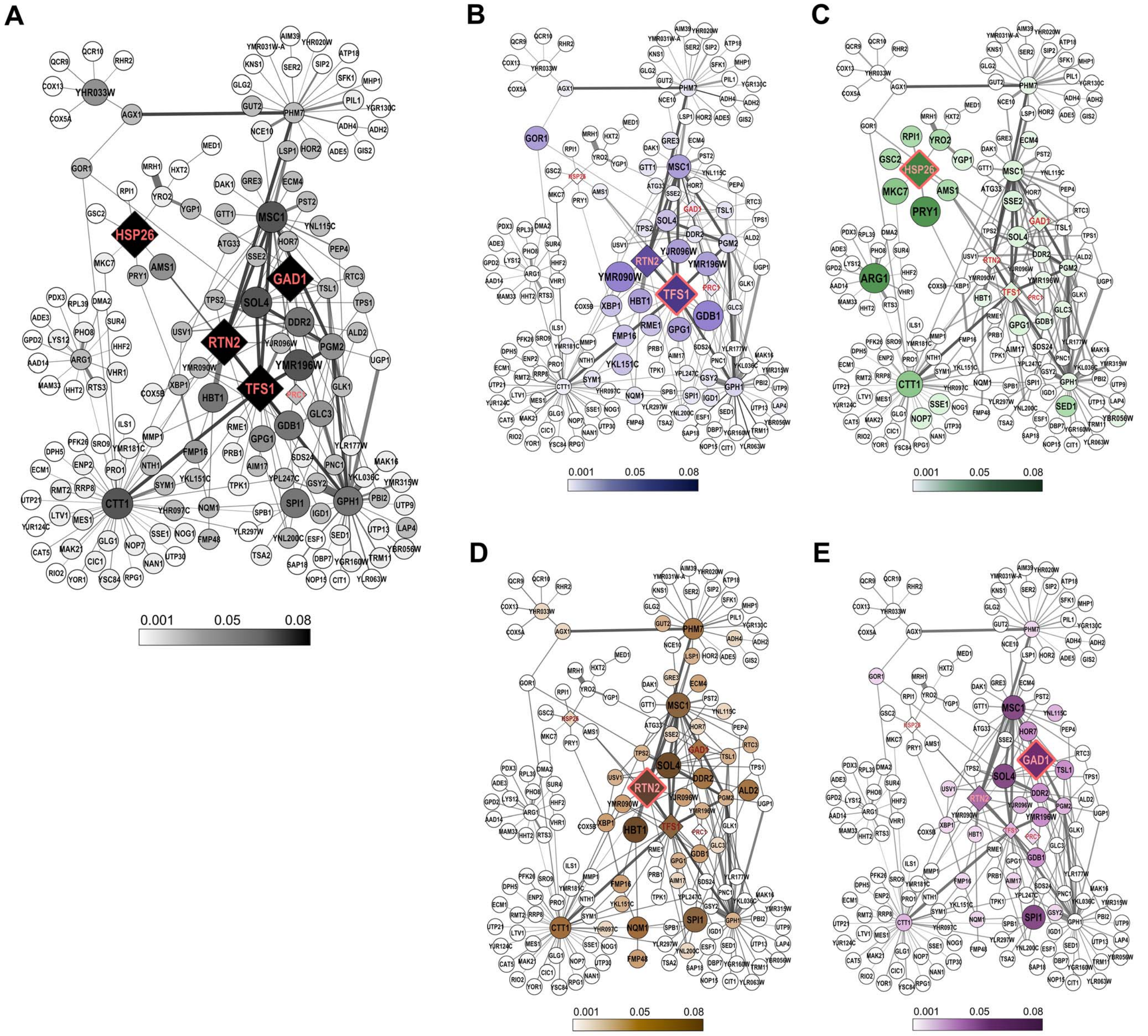

Figure 10. Yeast gene-subnetwork for DNA replication stress response and its SNP perturbation estimated by sparse CGGM. (A) The yeast subnetwork for DNA replication stress response and its direct/indirect perturbation by a SNP in the region of 1,095 kb on chromosome 4 estimated by sparse CGGM learning algorithm. This SNP directly perturbs *TFS1*, *HSP26*, *RTN2*, and *GAD1*, and the propagation of this direct perturbation to other parts of the network is obtained by performing inference on the estimated sparse CGGM. Edge thicknesses correspond to absolute values of edge weights in $\mathbf{\Theta}_{\mathbf{yy}}$. The diamond-shaped nodes represent gene-expression traits that are directly perturbed by the SNP, whereas the round and colored nodes represent those genes whose expressions are indirectly perturbed by the SNP. The color shade and size of nodes indicate the strength of the SNP perturbation of gene-expression trait. Our statistical framework allows the overall indirect SNP perturbation effects in Panel (A) to be decomposed into the components that arose from the propagation of the direct perturbation effects of each of (B) *TFS1*, (C) *HSP26*, (D) *RTN2*, and (E) *GAD1* by the given SNP.
doi:10.1371/journal.pcbi.1003420.g010

responsive protein has been found to have multi-stress response function [41]. Also, several hub genes in the subnetwork have been annotated as responding to other stress conditions such as heat shock (*HSP26*) and oxidative stress (*CTT1* and *GAD1*) [42–44].

Direct and Indirect SNP Perturbations of the Subnetwork. We examined the direct and indirect perturbations of this subnetwork by an eQTL located in 1,095 kb region of chromosome 4. In Figure 10A, the genes whose expressions are directly perturbed by this eQTL are shown as diamond-shaped nodes. The round and colored nodes correspond to genes whose expressions are indirectly perturbed by this eQTL. The node size and color shade correspond to the effect sizes of the eQTL, with larger and darker nodes for stronger SNP perturbations.

In Figure 10A, we see that only a small number of gene-expression traits (*TFS1*, *HSP26*, *GAD1*, *RTN2*, and *PRC1*) are directly perturbed by the given eQTL, whereas this direct eQTL effects are passed on to other parts of the network to affect the expressions of many other genes indirectly. These indirect eQTL effects tend to be stronger on the immediate neighbors of the directly perturbed genes and gradually decrease for those genes farther away from the directly perturbed genes.

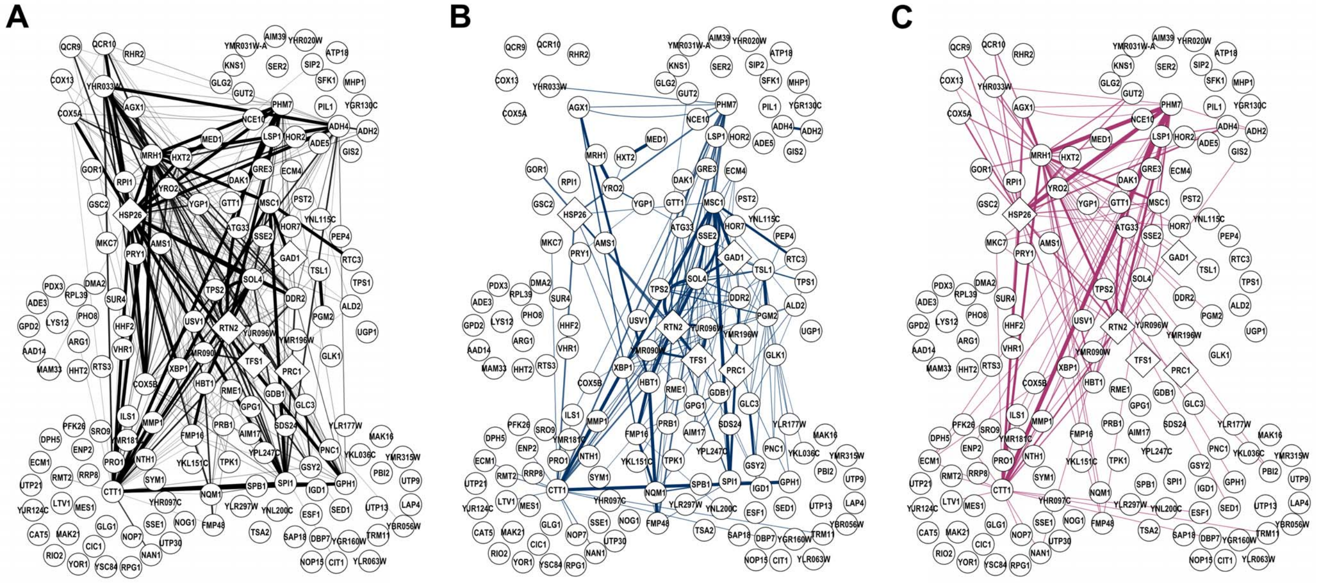

Figure 11. Decomposition of yeast gene-expression covariances for DNA replication stress response subnetwork using sparse CGGM. (A) The covariance of yeast gene expression data for the genes shown in Figure 10. Sparse CGGM allows the observed covariance in Panel (A) to be decomposed approximately into (B) the covariance induced by the gene network and (C) the covariance induced by SNP perturbations and its propagation through the network. Edge width corresponds to covariance or the strength of gene-gene interaction. We note that the edges show marginal dependencies in covariances rather than conditional dependencies in inverse covariances.
doi:10.1371/journal.pcbi.1003420.g011

Decomposition of SNP Perturbation Effects. While Figure 10A shows the overall perturbation effects of the given eQTL on the subnetwork, in order to obtain a more detailed description of the perturbation effects, we used Eq. (8) to perform a decomposition of the overall effects of this eQTL with respect to each of the directly-influenced genes from which the indirect eQTL effects originated. The results of the decomposition are shown for each of the four directly-influenced genes (*TFS1*, *HSP26*, *RTN2*, and *GAD1*) in Figures 10B–E, respectively. The genes whose expressions are directly perturbed by the eQTL and who pass such direct eQTL effects to other parts of the network are marked with red lines around the corresponding nodes in Figures 10B–E. Although *PRC1* is also directly perturbed by this eQTL, we do not show the result for *PRC1*, because the strength of the direct SNP perturbation on *PRC1* is significantly smaller than those for the other four directly-influenced genes. *PRC1* is connected to *TFS1* and its activity is known to be inhibited by *TFS1*-encoded protein with high affinity [45].

How strongly the direct eQTL perturbation effects propagate to other parts of the network depends on both the strengths of the direct SNP perturbations $\mathbf{\Theta_{xy}}$ and the strengths of network edge connections $\mathbf{\Theta_{yy}}$. For example, in Figure 10A, the given eQTL has the largest direct influence on *RTN2* among all of the directly-influenced genes, and as can be seen in Figure 10D, *RTN2* can pass on this direct SNP perturbation to a larger part of the network than *TFS1*, *HSP26*, and *GAD1*. On the other hand, as shown in Figures 10A and D, among *RTN2*'s neighbors, *HBT1* and *MSC1* receive the strongest indirect SNP perturbations because these two genes have stronger edge connections to *RTN2*.

Table 3. Prediction errors on yeast eQTL data.

	Sparse CGGM	GFlasso
Test-set Error	0.0343	0.1074

doi:10.1371/journal.pcbi.1003420.t003

Decomposition of Gene-Expression Covariance. Sparse CGGM also allows for a decomposition of the observed covariance of gene-expression data into two components, the covariance component induced by the gene network and the covariance component induced by the SNP perturbations, as described by Eq. (10). Based on the same subnetwork in Figure 10 and all eQTLs perturbing this subnetwork, we show the results of this decomposition in Figure 11. Figure 11A represents the observed covariance in the gene-expression data (the left-hand side of Eq. (10)), and Figures 11B and C show the covariance component induced by the gene network (the first term on the right-hand side of Eq. (10)), and the covariance component induced by eQTLs (the second term on the right-hand side of Eq. (10)), respectively. Edge thicknesses indicate the absolute values of covariances. We note that the edges in Figure 11 represent marginal dependencies in covariances, whereas the edges in Figure 10 represent conditional dependencies between two gene-expression traits conditional on all the other gene-expression traits.

Experimental Evidence for the Estimated Subnetwork. Although the DNA replication stress response subnetwork in Figures 10 and 11 was obtained computationally using our proposed statistical method, we found that this computational result is supported well by the results from experimental perturbation study in the literature and that many genes in the subnetwork have a known role in DNA replication stress response [46]. In [46], 45 up-regulated transcripts were identified in a telomerase-deficient mutant, after an exposure to telomere shortening and cellular senescence. Out of these 45 genes, 32 genes appeared in our gene-expression dataset of 3,684 genes, and 30 out of the 32 genes were one or two edges away from *TFS1* in our subnetwork in Figure 10A: 13 genes were immediate neighbors of *TFS1* and another 17 genes were neighbors of either *CTT1* (cytosolic atlas T) or *DDR2* (DNA damage responsive). Both *CTT1* and *DDR2* were neighbors of *TFS1* in our subnetwork and

are known multi-stress response proteins targeted by the transcription activator *MSN2* that responds to stress conditions [42]. In addition, our subnetwork captures many of the known interactions among several key elements in DNA replication stress response, such as *PHM7*, *SOL4*, *RTN2*, and *PGM2* [40,47].

Finally, our results indicate that different types of stress response pathways are closely related to each other, as genes involved in stress responses under conditions other than DNA replication are often found as neighbors of DNA replication stress response genes in our subnetwork. For example, *HSP26*, a heat shock protein with chaperon activity, is another target of *MSN2*, and is known to be involved in response to heat shock and other stimuli [48]. This *HSP26* gene is connected in our subnetwork to *XBP1*, a transcriptional repressor whose relative distribution in the nucleus increases under DNA replication stress [40]. Likewise, *GAD1*, a glutamate decarboxylase, is required in oxidative stress tolerance [44] and is a neighbor of *TFS1* and *RTN2* in our subnetwork.

Overall, we conclude that the gene network obtained computationally by sparse CGGM learning algorithm using SNPs as naturally-occurring perturbations of the gene regulatory system generally matches the network obtained from experimental perturbation study.

Prediction Accuracy for Yeast Gene Expressions Given SNPs. As a quantitative evaluation of our method on yeast eQTL dataset, we computed prediction errors on an independent test set for sparse CGGM and compared the result with what we obtained for GFlasso (Table 3). While it took sparse CGGM and GFlasso less than a day for a single run of model training on all SNPs and gene-expression traits, for MRCE a single run of the approximate learning method was not completed after several weeks of computation and we were unable to obtain prediction errors for MRCE. We estimated a model on 100 samples and computed prediction errors on the test set of the remaining 14 samples. During the estimation stage, we selected the optimal regularization parameters by cross-validation with three random splits of the 100 samples into 90 samples for fitting a model and 10 samples for computing cross-validation errors. Since GFlasso requires a known gene network, we used the correlation coefficient matrix of gene-expression data thresholded at 0.6 as an input gene network. In visual inspection, we found that this thresholded correlation matrix for gene network captured gene module structures reasonably well, when compared to the results from hierarchical agglomerative clustering applied to the gene-expression data. The results in Table 3 show that sparse CGGM outperforms GFlasso on predicting gene-expression levels from SNP genotypes.

Discussion

In this paper, we presented a new statistical framework for genetical genomics analysis to learn a gene network by treating SNPs as naturally occurring perturbants of a gene network. Within this framework, we introduced a statistical model, called a sparse CGGM, for modeling a gene network under SNP perturbations and discussed an efficient learning algorithm and inference methods. While genetical genomics approach has been recognized as a more effective and less costly method for learning a gene network than experimental methods, this approach has not been widely used mainly because of the computational challenge that the effects of perturbations by often millions genetic variants at a time need to be decoded from data. Our approach directly addresses this challenge and identifies a gene network by decoupling the effects of multifactorial perturbations in eQTL data. At the same time, our approach addresses many of the weaknesses of the experimental methods and is able to identify which genes are directly perturbed by each SNP or are indirectly perturbed as downstream effects in the pathway. As eQTL data collection is being routinely performed for model organisms, and is more amenable for human tissues than experimental perturbations, our approach opens up doors to the possibility of leveraging these datasets for gene network learning rather than focusing on finding eQTLs from such data. Although the primary goal of our work and more generally genetical genomics analysis is to identify a gene network, our statistical approach has additional advantages of enhancing the current statistical tools of eQTL mapping and extracting significantly more detailed information on the functional role of eQTLs in the context of gene network.

Our approach provides a flexible statistical framework for learning a gene network along with eQTLs that can be easily extended in several different ways. For example, although in this paper, our gene network was defined over the expression levels of mRNAs, it is straightforward to include microRNA expression data to construct a network over both mRNA and microRNA expressions, both of which can be perturbed by genetic variants. Another possible extension is to model epistatic interactions among SNPs within sparse CGGM by introducing additional features for SNP interactions in the probabilistic graphical model.

However, there are certain limitations to our approach. Although our approach can handle thousands of gene-expression traits and SNPs efficiently, it is still not efficient enough to be directly applied to genome-wide analysis of eQTL datasets of higher-level organisms with tens of thousands of gene-expression traits and millions of SNPs. For such large-scale datasets, we suggest to split the full dataset into smaller sets of gene-expression traits by applying a clustering algorithm to obtain coarse-grained gene modules. Then, our approach can be applied to each subset of gene-expression traits for coarse-grained gene modules to extract fine-grained information on gene-network connectivities. In order to perform a full joint analysis of all data, in future work, we will consider improving the computational bottleneck of matrix inversion for the gene network parameters in the learning algorithm by replacing it with an approximate but computationally less expensive inversion.

Another future direction is to relax the assumption in our model that the gene-expression traits under SNP perturbations follow a Gaussian distribution. Although Gaussian graphical models have been widely used to infer a gene network from gene-expression data due to many of the properties of Gaussian distributions that lead to easy computations, this assumption can be potentially restrictive for modeling realistic biological processes.

The software for sparse CGGMs is available at http://www.cs.cmu.edu/~sssykim/softwares/softwares.html#scggm.

Supporting Information

Text S1 Proof of convexity of sparse CGGM optimization problem.
(PDF)

Text S2 Learning algorithm for sparse CGGMs.
(PDF)

Acknowledgments

We thank Kyung-Ah Sohn for the help with preliminary results. We thank Joel McManus for helpful discussion on yeast data analysis.

Author Contributions

Conceived and designed the experiments: SK. Performed the experiments: LZ. Analyzed the data: SK LZ. Contributed reagents/materials/analysis tools: SK LZ. Wrote the paper: SK.

References

1. Tong A, Evangelista M, Parsons A, Xu H, Bader G, et al. (2001) Systematic genetic analysis with ordered arrays of yeast deletion mutants. Science 294: 2364–2368.
2. Hu Z, Killion P, Iyer V (2007) Genetic reconstruction of a functional transcriptional regulatory network. Nature Genetics 39: 683–687.
3. Chua G, Morris Q, Sopko R, Robinson M, Ryan O, et al. (2006) Identifying transcription factor functions and targets by phenotypic activation. PNAS 103: 12045–50.
4. Jansen RC, Nap JP (2001) Genetical genomics: the added value from segregation. Trends in Genetics 17: 388–391.
5. Jansen R (2003) Studying complex biological systems using multifactorial perturbation. Nature Reviews Genetics 4: 145–151.
6. Morley M, Molony C, Weber T, Devlin J, Ewens K, et al. (2004) Genetic analysis of genome-wide variation in human gene expression. Nature 430: 743–747.
7. Stranger B, Forrest M, Clark A, Minichiello M, Deutsch S, et al. (2005) Genome-wide associations of gene expression variation in humans. PLoS Genetics 1: e78.
8. Kim S, Xing E (2009) Statistical estimation of correlated genome associations to a quantitative trait network. PLoS Genetics 5: e1000587.
9. Logsdon B, Mezey J (2010) Gene expression network reconstruction by convex feature selection when incorporating genetic perturbations. PLoS Computational Biology 6: e1001014.
10. Chaibub N, Keller M, Attie A, Yandell B (2010) Causal graphical models in systems genetics: a unified framework for joint inference of causal network and genetic architecture for correlated phenotypes. Annals of Applied Statistics 4: 320–339.
11. Aten J, Fuller T, Lusis A, Horvath S (2008) Using genetic markers to orient the edges in quantitative trait networks: the NEO software. BMC Systems Biology 2: 320–339.
12. Ye C, Galbraith S, Liao J, Eskin E (2008) Using network component analysis to dissect regulatory networks mediated by transcription factors in yeast. PLoS Computational Biology 5: e1000311.
13. Lee SI, Dudley A, Drubin D, Silver P, Krogan N, et al. (2009) Learning a prior on regulatory potential from eQTL data. PLoS Genetics 5: e1000358.
14. Meinshausen N, Buhlmann P (2006) High-dimensional graphs and variable selection with the lasso. The Annals of Statistics 34: 1436–1462.
15. Schafer J, Strimmer K (2005) A shrinkage approach to large-scale covariance matrix estimation and implications for functional genomics. Statistical Applications of Genetics and Molecular Biology 4: 32.
16. Friedman J, Hastie T, Tibshirani R (2008) Sparse inverse covariance estimation with the graphical lasso. Biostatistics 9: 432–41.
17. Banerjee O, Ghaoui LE, dAspremont A (2008) Model selection through sparse maximum likelihood estimation for multivariate Gaussian or binary data. Journal of Machine Learning Research 9: 485–516.
18. Shi W, Lee K,Wahba G (2007) Detecting disease causing genes by LASSO-Patternsearch algorithm. Technical Report 1140, Department of Statistics, University of Wisconsin.
19. Wu TT, Chen YF, Hastie T, Sobel E, Lange K (2009) Genome-wide association analysis by lasso penalized logistic regression. Bioinformatics 25: 714–721.
20. The International HapMap 3 Consortium (2010) Integrating common and rare genetic variation in diverse human populations. Nature 467: 52–58.
21. Brem R, Kruglyak L (2005) The landscape of genetic complexity across 5,700 gene expression traits in yeast. PNAS 102: 1572–1577.
22. Rothman A, Levina E, Zhu J (2010) Sparse multivariate regression with covariance estimation. Journal of Computational and Graphical Statistics 19: 947–962.
23. Yin J, Li H (2011) A sparse conditional Gaussian graphical model for analysis of genetical genomics data. Annals of Applied Statistics 5: 2630–50.
24. Banerjee O, El Ghaoui L, d'Aspremont A (2008) Model selection through sparse maximum likeli- hood estimation for multivariate Gaussian or binary data. Journal of Machine Learning Research 9: 485–516.
25. Tibshirani R (1996) Regression shrinkage and selection via the lasso. Journal of Royal Statistical Society, Series B 58: 267–288.
26. Friedman J, Hastie T, Tibshirani R (2007) Pathwise coordinate optimization. The Annals of Applied Statistics 1: 302–332.
27. Li B, Chun H, Zhao H (2012) Sparse estimation of conditional graphical models with application to gene networks. Journal of the American Statistical Association 107: 152–167.
28. Mardia K, Kent J, Bibby J (1979) Multivariate Analysis. Academic Press.
29. Koller D, Friedman N (2009) Probabilistic Graphical Models: Principles and Techniques. MIT Press.
30. Boyd S, Vandenberghe L (2004) Convex Optimization. Cambridge University Press.
31. Nesterov Y (2005) Smooth minimization of non-smooth functions. Math Program 103: 127–152.
32. Beck A, Teboulle M (2009) A fast iterative shrinkage-thresholding algorithm for linear inverse problems. SIAM Journal of Imaging Sciences 2: 183–202.
33. Nesterov Y (2007) Gradient methods for minimizing composite objective function. CORE Dis- cussion Papers 2007076, Universit catholique de Louvain, Center for Operations Research and Econometrics (CORE).
34. Chen X, Lin Q, Kim S, Carbonell J, Xing E (2012) Smoothing proximal gradient method for general structured sparse regression. Annals of Applied Statistics 6: 719–752.
35. Luxburg U (2007) A tutorial on spectral clustering. Statistics and Computing 17: 395–416.
36. Chakrabarti D, Faloutsos C (2006) Graph mining: Laws, generators, and algorithms. ACM Computing Surveys 38: 2.
37. Zhang B, Horvath S (2005) A general framework for weighted gene co-expression network analysis. Statistical Applications in Genetics and Molecular Biology 4: Article 17.
38. Zhu J, Zhang B, Smith E, Drees B, Brem R, et al. (2008) Integrating large-scale functional genomic data to dissect the complexity of yeast regulatory networks. Nature Genetics 40: 854–61.
39. Robinson L, Tatchell K (1991) TFS1: A suppressor of cdc25 mutations in Saccharomyces cerevisiae. Molecular and General Genetics 230: 241–250.
40. Tkach JM, Yimit A, Lee AY, Riffle M, Costanzo M, et al. (2012) Dissecting DNA damage response pathways by analysing protein localization and abundance changes during DNA replication stress. Nature Cell Biology 14: 966–976.
41. Kobayashi N, Mcclanahan TK, Simon JR, Treger JM, Mcentee K (1996) Structure and functional analysis of the multistress response gene DDR2 from Saccharomyces cerevisiae. Biochemical and Biophysical Research Communications 229: 540–547.
42. Schmitt AP, McEntee K (1996) Msn2p, a zinc finger DNA-binding protein, is the transcriptional activator of the multistress response in Saccharomyces cerevisiae. PNAS 93: 5777–82.
43. Grant CM, Perrone G, Dawes IW (1998) Glutathione and catalase provide overlapping defenses for protection against hydrogen peroxide in the yeast Saccharomyces cerevisiae. Biochemical and Biophysical Research Communications 253: 893–898.
44. Coleman S, Fang T, Rovinsky S, Turano F, Moye-Rowley W (2000) Expression of a glutamate decarboxylase homologue is required for normal oxidative stress tolerance in Saccharomyces cerevisiae. Journal of Biological Chemistry 276: 244–250.
45. Bruun AW, Svendsen I, Sorensen SO, Kielland-Brandt MC, Winther JR (1998) A high-affinity inhibitor of yeast carboxypeptidase Y is encoded by TFS1 and shows homology to a family of lipid binding proteins. Biochemistry 37: 3351–3357.
46. Teng SC, Epstein C, Tsai YL, Cheng HW, Chen HL, et al. (2002) Induction of global stress response in Saccharomyces cerevisiae cells lacking telomerase. Biochemical and Biophysical Research Communications 291: 714–721.
47. Zhu J, Sova P, Xu Q, Dombek KM, Xu EY, et al. (2012) Stitching together multiple data dimensions reveals interacting metabolomic and transcriptomic networks that modulate cell regulation. PLoS Biology 10: e1001301.
48. Amoros M, Estruch F (2001) Hsf1p and Msn2/4p cooperate in the expression of Saccharomyces cerevisiae genes HSP26 and HSP104 in a gene- and stress type-dependent manner. Molecular Microbiology 39: 1523–1532.
